# Four-Octyl itaconate ameliorates periodontal destruction via Nrf2-dependent antioxidant system

**DOI:** 10.1038/s41368-022-00177-1

**Published:** 2022-05-31

**Authors:** Liangjing Xin, Fuyuan Zhou, Chuangwei Zhang, Wenjie Zhong, Shihan Xu, Xuan Jing, Dong Wang, Si Wang, Tao Chen, Jinlin Song

**Affiliations:** 1 College of Stomatology, Chongqing Medical University, Chongqing Key Laboratory for Oral Diseases and Biomedical Sciences, Chongqing Municipal Key Laboratory of Oral Biomedical Engineering of Higher Education, Chongqing, China; 2 Department of Ultrasound, The First Affiliated Hospital, Chongqing Medical University, Chongqing, China

**Keywords:** Periodontitis, Molecular medicine

## Abstract

Periodontitis is a widespread oral disease characterized by continuous inflammation of the periodontal tissue and an irreversible alveolar bone loss, which eventually leads to tooth loss. Four-octyl itaconate (4-OI) is a cell-permeable itaconate derivative and has been recognized as a promising therapeutic target for the treatment of inflammatory diseases. Here, we explored, for the first time, the protective effect of 4-OI on inhibiting periodontal destruction, ameliorating local inflammation, and the underlying mechanism in periodontitis. Here we showed that 4-OI treatment ameliorates inflammation induced by lipopolysaccharide in the periodontal microenvironment. 4-OI can also significantly alleviate inflammation and alveolar bone loss via Nrf2 activation as observed on samples from experimental periodontitis in the C57BL/6 mice. This was further confirmed as silencing Nrf2 blocked the antioxidant effect of 4-OI by downregulating the expression of downstream antioxidant enzymes. Additionally, molecular docking simulation indicated the possible mechanism under Nrf2 activation. Also, in Nrf2^−/−^ mice, 4-OI treatment did not protect against alveolar bone dysfunction due to induced periodontitis, which underlined the importance of the Nrf2 in 4-OI mediated periodontitis treatment. Our results indicated that 4-OI attenuates inflammation and oxidative stress via disassociation of KEAP1-Nrf2 and activation of Nrf2 signaling cascade. Taken together, local administration of 4-OI offers clinical potential to inhibit periodontal destruction, ameliorate local inflammation for more predictable periodontitis.

## Introduction

Initiation of periodontitis and its propagation occur mainly due to dysbiosis of the oral microbiota (dental biofilm), which induces a local inflammatory response and stimulates the innate immune response first. This dysbiosis progressively destroys the tooth-supporting tissues, subsequently loosening teeth and eventually their loss.^1–3^ Epidemiologically periodontitis is associated with other chronic inflammation-driven disorders, such as diabetes, atherosclerotic disease, Alzheimer disease, and certain cancers.^4–7^ Management of periodontitis can improve the prognosis of systemic diseases,^5,8^ so the treatment of periodontitis should be considered and planned carefully allowing a constant evolution for the betterment of patient outcomes. Managements currently available for periodontitis include nonsurgical therapy (e.g., tooth scaling, periodontal pocket flush, and root planning) and regenerative surgery (e.g., guided tissue regeneration) performed to regenerate the periodontal tissue.^9^ Additionally, adjunctive drugs containing antibiotics and antimicrobials are applied to the pocket for topical periodontal treatment.^1^ However, the outcomes of the above procedures are variable and unsatisfactory due to the persistent inflammation and the activated innate immune response that characterize periodontitis.^10,11^ So, a high-efficiency therapy needs to meet the requirements that it can both alleviate local inflammatory damage and modulate the innate immune response.

Macrophages have been demonstrated to be a prerequisite for periodontal regeneration through a shift in polarization phenotypes.^5^ In addition, damage-associated molecular patterns (DAMPs), arising from the altered innate immune response in periodontal tissues,^12^ directly affect the metabolic adaptation of macrophages. And pathogen-associated molecular patterns (PAMPs) induce the permanent pro-inflammatory phenotype of macrophages and indirectly activate adaptive immune responses.^13,14^ Therefore, it is crucial to explore the biological behavior of macrophages to elucidate the mechanism of periodontal tissue regeneration.

As one of the DAMPs, reactive oxygen species (ROS) are considered to be a mixed blessing, playing an important role in both physiological and pathological metabolism.^15,16^ At the physiological level, ROS act as second messengers for cell signaling, gene regulation, and immune defense.^17,18^ However, the overproduction of ROS initiates an oxidative imbalance together with an impaired antimicrobial capacity that triggers irreversible tissue injury.^17,19^ Currently, more attention is being paid to the role of ROS in establishing a microenvironment that underlies the pathogenesis of chronic inflammatory conditions.^16,20,21^ Overproduction of ROS has deleterious effects on host cells via processes such as lipid peroxidation or DNA damage.^22^ This is considered to be one of the most important issues associated with the progression of periodontitis and increased attachment loss.^23^

Considering the aforementioned issues, new therapeutic strategies have now been focused on minimizing the deleterious effects arising from ROS. Through redox-sensitive signaling cascade, Nuclear factor E2-related factor 2 (Nrf2) can upregulate the expression of antioxidant response element (ARE), which provides cell protection and against various oxidative injuries.^24^ Under physiological conditions, it localizes in the cytoplasm and binds to Kelch-like ECH associated protein 1 (KEAP1), which induces Nrf2 protein degradation through the CUL3 ubiquitin-proteasome system.^25^ When oxidative stress occurs, Nrf2 is disassociated from KEAP1-Nrf2 complex and translocates to the cell nuclei, regulating the transcription of ARE-dependent genes and various detoxifying enzymes.^17,25^ Therefore, the emergence of Nrf2 activators could provide new therapeutic drug options to alleviate oxidative stress and improve periodontal treatments.

Findings from recent studies have reported an emerging molecule, four-octyl itaconate (4-OI) that activates Nrf2 pathway by alkylating KEAP1’s key cysteine residues to disassociate KEAP1-Nrf2, has been utilized for controlling many inflammatory diseases, such as sepsis and acute lung injury.^26–28^ Intracellularly, esterase and lipopolysaccharide (LPS)-activated macrophages hydrolyze 4-OI to form itaconate, making it a cell-permeable substitution for itaconate.^26^ However, the potential activity of 4-OI in the treatment of periodontitis has never been studied. Herein, we report, for the first time, the application of 4-OI in the treatment of periodontitis, and investigate the effect of 4-OI on activation of Nrf2 cascade to prevent periodontal inflammation and alveolar bone loss through in vivo and in vitro experiments.

## Results

### Local administration of 4-OI ameliorates periodontal inflammation and alveolar bone loss in vivo

**Fig. 1:**
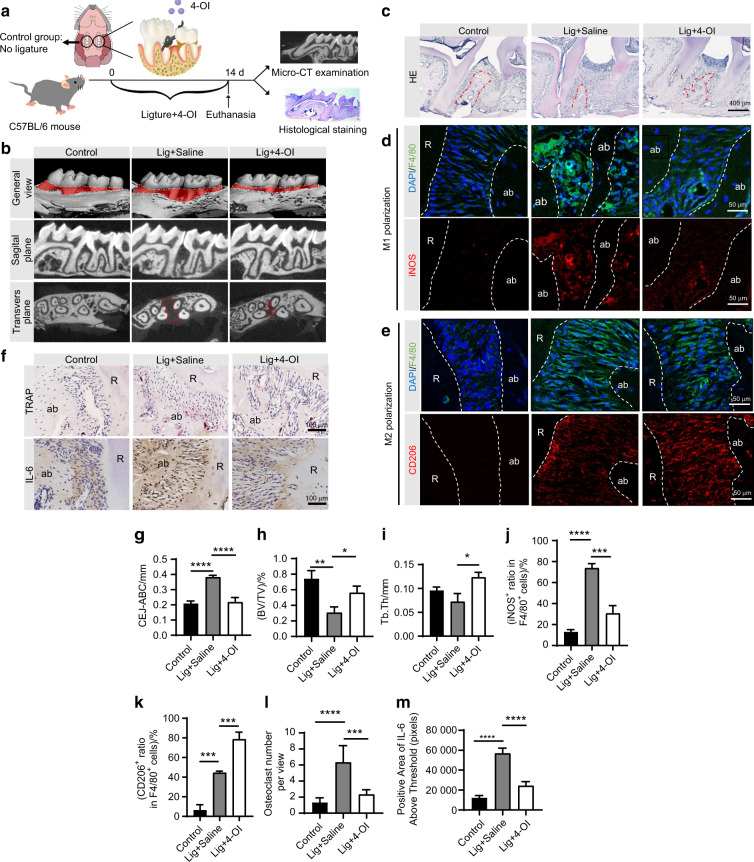
Local administration of 4-OI ameliorates periodontal inflammation and alveolar bone loss in vivo. **a** Timeline for the operation process in C57BL/6 male mice. **b** Micro-CT image of the alveolar bone. The red part displayed the exposure of root. **c** Representative images of H&E staining of the periodontitis areas. (Scale bar = 400 μm). **d, e** IF staining of M1 polarization-related markers (iNOS/F4/80) and M2 polarization-related markers (CD206/F4/80) and in macrophages across the periodontal region at 14 days post-operation. (Scale bar = 50 μm). **f** Representative images of TRAP staining and the IHC staining of IL-6 in the periodontal tissue. (Scale bar = 100 μm). **g** CEJ-ABC distance. **h** BV/TV. **i** Tb.Th. **j, k** Semi-quantitative analyses of iNOS^+^ ratio in F4/80^+^ cells and CD206^+^ ratio in F4/80^+^ cells. **l** Quantitative analysis of osteoclast number per view at alveolar bone. **m** Positive area of IL-6 above threshold. Values are presented as mean ± SD. *P < 0.05, **P < 0.01, ***P < 0.001, ****P < 0.000 1. Lig ligature, ab alveolar bone, R root

To validate whether 4-OI can protect against ligature-induced experimental periodontitis, C57BL/6 mice with experimental periodontitis were either injected locally with or without 4-OI daily for 14 days (Fig. 1a). In clinic, the cementoenamel junction and alveolar bone crest (CEJ-ABC) distance is an important indicator of periodontal attachment loss, which we used to quantify the degree of periodontal bone resorption (Fig. 1b, g). Meanwhile, we also selected bone volume per tissue volume (BV/TV) percentage and trabecular thickness (Tb.Th) to determine the variances in bone volume density. The Lig + Saline group showed significant alveolar bone resorption compared with the control group. However, the adverse trend of alveolar bone resorption was alleviated by 4-OI (Fig. 1b, h). Additionally, trabecular features were substantially improved after 4-OI treatment (Fig. 1b, i).

Hematoxylin and eosin (H&E) staining indicated 4-OI could play a pivotal role in decreasing alveolar bone loss in experimental periodontitis models (Fig. 1c). Next, surface markers and soluble mediators (iNOS for M1 macrophages, CD206 for M2 macrophages) plus F4/80 were used to identify the polarization states of recruited macrophages in the periodontal region (between the maxillary first molar and second molar) during the operation process. Immunofluorescent (IF) staining and quantitative analysis revealed an increasing number of CD206^+^/F4/80^+^ cells in the slices after 4-OI treatment (Fig. 1e, k), while more iNOS^+^/ F4/80^+^ cells were found in the Lig + Saline group than the Lig + 4-OI group at 14 days post-operation (Fig. 1d, j). Compared with the control group, increased osteoclasts were examined in the Lig + Saline group. Noticeably, 4-OI treatment practically decreased osteoclasts in the alveolar bone of ligature-induced periodontitis mice (Fig. 1f, l). The IL-6 expression levels were markedly lower in the Lig + 4-OI group than in the Lig + Saline group, suggesting that 4-OI effectively controlled the progression of inflammation in periodontal tissues (Fig. 1f, m, S1). Altogether, we surmise that 4-OI may play a key role in ameliorating inflammatory damage and inhibiting periodontal destruction.

### 4-OI induces a shift in the polarization phenotype of macrophages in vitro

**Fig. 2:**
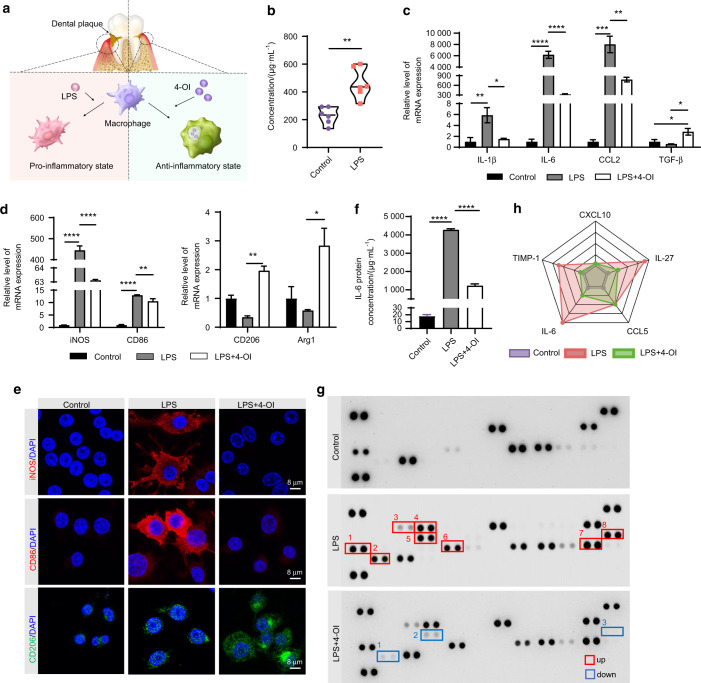
4-OI induces a shift in polarization phenotype of macrophages in vitro. **a** Schematic illustration for the method utilized for the local administration of 4-OI in a periodontitis model. **b** The concentration of itaconate in RAW 264.7 analyzed using UPLC-MS assay. **c, d** Expression of M1 and M2-related genes in RAW 264.7 using qRT-PCR (The M1-related IL-1β, IL-6, CCL2, iNOS, CD86, and the M2-related TGF-β, Arg-1 and CD206). **e** Micrographs showing staining of iNOS, CD86 and CD206 obtained using confocal laser scanning microscopy. (Nucleus: blue, iNOS and CD86: red, CD206: green. Scale bar = 8 μm). **f** IL-6 protein concentration of RAW 264.7 tested by ELISA assay. **g** The cytokine array images of RAW 264.7. Each two adjacent dots represent one kind of cytokine, among which the representative upregulated (up) cytokines of the LPS group compared to the control group and downregulated (down) cytokines of the LPS + 4-OI group compared to the LPS group were marked in different color boxes (red and blue). **h** The expression profiles of differentially expressed factors in panel **g** shown as a Radar Chart. All data are presented as the mean ± SD. *P < 0.05, **P < 0.01, ***P < 0.001, ****P < 0.000 1

Macrophages were treated with 4-OI and their polarization status and expression of inflammation-related factors were examined (Fig. 2a). As shown in Fig. 2b, itaconate reached a significant level of (462.6 ± 51.51) μg·mL^−1^ in macrophages after LPS stimulation, as compared with the expression in the control group (^**^P < 0.01), indicating that itaconate is a metabolite in response to inflammatory stimuli. Our data showed that a high dose of 4-OI led to significant inhibition of cellular activity (Fig. S2), and we chose a sufficient concentration (200 μmol·L^−1^) for cell proliferation in subsequent experiments. To validate the effects of 4-OI on LPS-induced inflammatory state, the expression of specific inflammatory-related cytokines on RAW264.7 and bone-marrow-derived macrophages (BMDMs) were analyzed. According to the qRT-PCR results, 4-OI effectively suppressed the expression levels of M1-related factors such as IL-1β, IL-6, and CCL2 on both RAW264.7 (Fig. 2c) and BMDMs (Fig. S3), while the expression of M2-related factors including TGF-β was enhanced in 4-OI treated RAW264.7 (Fig. 2c). The expression levels of M1-phenotype surface markers iNOS and CD86, were significantly decreased in the LPS + 4-OI group (Figs. 2d, S3). Moreover, the expression levels of M2 markers CD206 and Arg-1, in macrophages treated with 4-OI were observed to be higher than in the LPS group, suggesting that 4-OI exerts a regulatory role in controlling inflammation and polarization (Figs. 2d, S3). The IF staining results of iNOS, CD86, and CD206 were in line with the above trends (Figs. 2e, S4). The fluorescence intensity was measured by semi-quantitative analysis (Fig. S5). These results were further confirmed using ELISAs that showed lower levels of IL-6 under 4-OI treatment (Fig. 2f). It is reported that the reversal of the pro-inflammatory state and the altering patterns of exocytotic cytokines are inextricably linked.^29,30^ Therefore, we conducted an antibody array assay on mouse cytokines to analyze the change after 4-OI treatment (Fig. 2g). Relative to the control group, the results showed that the levels of pro-inflammatory factors were significantly elevated after LPS stimulation (Fig. 2g, Table S3). As expected, compared to the LPS group, overt lower levels of pro-inflammatory factors, such as IL-6, IL-27, and TIMP-1, were observed in the LPS + 4-OI group. According to the data readout, a radar chart (Fig. 2h) analysis was carried out to highlight the integrated changes in cytokine expression. The results were in line with the above outcomes, and especially the downregulation of IL-6 and IL-27 indicated that 4-OI is effective in shutting down inflammation and regulating the polarization of macrophages from M1 to M2 phenotype.

### 4-OI scavenges excess ROS and mitigates oxidative damage in vitro

**Fig. 3:**
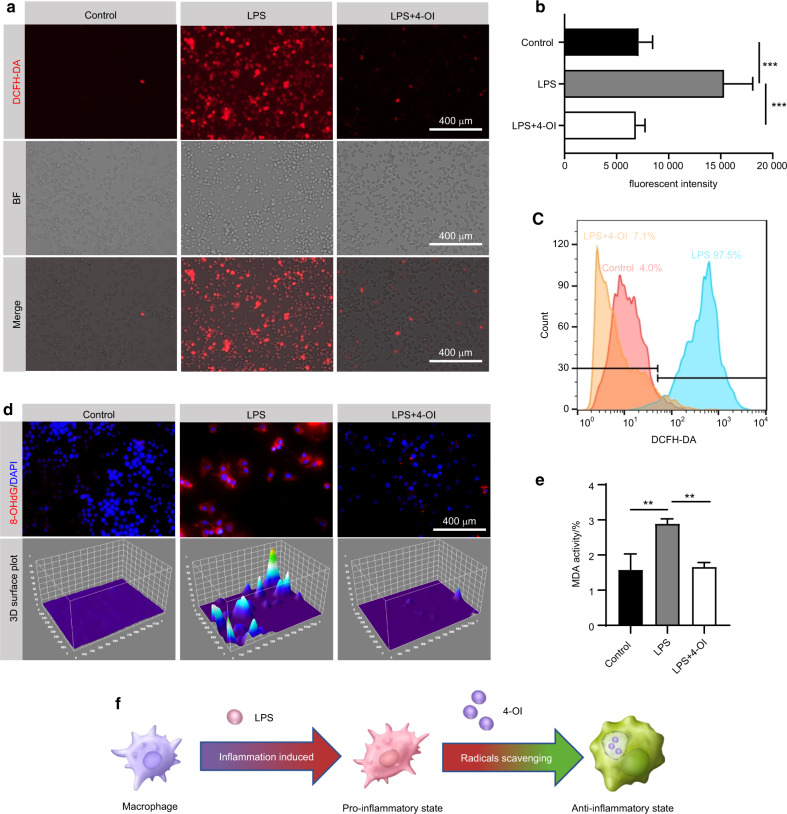
4-OI scavenges excess ROS and mitigates oxidative damage in vitro. Fluorescent images (**a**) and semi-quantitative analysis (**b**) of the intracellular ROS in RAW 264.7 measured by DCFH-DA staining. (Scale bar = 400 µm). **c** Flow cytometry images of intracellular ROS in RAW264.7 stained by DCFH-DA. **d** Representative fluorescent images and 3D surface plot images of 8-OHdG and nuclei in RAW 264.7. (Nucleus: blue, 8-OHdG: red. Scale bar = 400 µm). **e** The MDA content of RAW 264.7. **f** Schematic diagram of applying 4-OI to reduce LPS-induced oxidative damage in vitro. Data are expressed as the mean ± SD. *P < 0.05, **P < 0.01, ***P < 0.001, ****P < 0.000 1

It has been demonstrated that oxidative stress played a negative role in the progression of periodontitis.^16^ To further unravel the underlying mechanisms of 4-OI against oxidative stress, we examined the levels of ROS, MDA, 8-OHdG, and the activities of SOD and CAT in macrophages. Intracellular ROS measured by DCFH-DA staining showed that ROS accumulated in RAW264.7 (Fig. 3a) and BMDMs (Fig. S6) after LPS treatment and pretreatment with 4-OI predominantly decreased ROS accumulation. DCFH-DA fluorescence intensity is shown in Fig. 3b. A similar downward trend of intracellular ROS in RAW264.7 was observed by flow cytometry analysis in response to 4-OI stimulation (Fig. 3c). Meanwhile, macrophages exposed to LPS also exhibited a marked elevation of 8-OHdG, which is a biomarker of DNA damage induced by oxidative stress. However, 8-OHdG expression was downregulated by 4-OI (Figs. 3d, S4). Simultaneously, the levels of MDA were significantly increased after LPS exposure, but 4-OI treatment prevented the MDA elevation (Fig. 3e). In addition, the reduced enzyme activity of SOD and downregulated protein expression of CAT, suggested that 4-OI exerted antioxidant effects (Figs. S7, 8). Therefore, 4-OI scavenges excessive ROS, and could effectively regulate the redox balance in the inflammatory periodontal microenvironment, thereby, reducing the oxidative damage to host cells (Fig. 3f).

### 4-OI attenuates oxidative damage via disassociation of KEAP1-Nrf2 complex in vitro

**Fig. 4:**
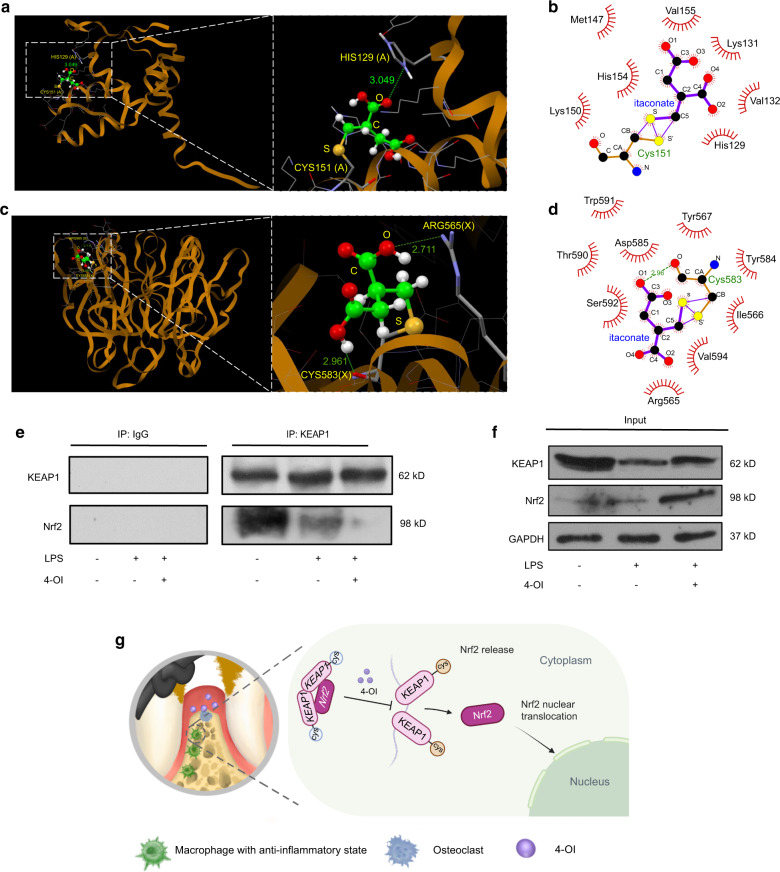
4-OI attenuates oxidative damage via disassociation of KEAP1-Nrf2 complex in vitro. **a** View and zoomed in view of the binding modes of itaconate with BTB domain in binding site of KEAP1 (PDB code: 5DAF). The itaconate molecule structure is displayed in Ball and sticks style and capped sticks are the amino acids of BTB domain in KEAP1. Green dashed lines are hydrogen bonds. **b** 2D interaction diagram depicting the interaction pattern of itaconate with BTB domain. **c** View and zoomed in view of the binding modes of itaconate with residue Cys583 in Kelch domain in binding site of KEAP1 (PDB code: 2FLU). **d** 2D interaction diagram depicting the interaction pattern of itaconate with residue Cys583 in Kelch domain. **e** Co-IP assay in RAW264.7. **f** Expression of listed proteins in cytosol fraction lysates tested by western blotting. **g** Schematic illustration of the therapeutic role of 4-OI in the inflammatory periodontal tissue

Nrf2 is a central response element against oxidative stress, playing a crucial role in mitigating ROS and cell damage.^24^ We first verified whether 4-OI can facilitate the disassociation of KEAP1-Nrf2 complex in LPS-induced macrophages. Studies have demonstrated that 4-OI is hydrolyzed to form itaconate when it penetrates into the activated macrophages.^26^ In an effort to model the itaconate on binding to BTB domain and Kelch domain in KEAP1 protein, molecular docking was carried out. The docking scores of cysteine residues with itaconate were collected in Table 1. In our docking model, the unsaturated alkene double bond of itaconate covalently bonds to the reactive sulfur atom of Cys151 in KEAP1-BTB domain (PDB: 5DAF) by Michael addition (Fig. 4a). Itaconate has a stable interaction with Cys155 via one hydrogen bond (~3.049 Å) between one of the carbonyl oxygen atoms with His129 residue (Fig. 4a). As shown in Fig. 4b, itaconate interacted hydrophobically with His129, Val132, Lys131, Met147. Lys150, His154, and Val155. In the meantime, we also examined the interaction of itaconate at the Kelch domain of KEAP1. The residues Arg565 and Cys583 formed two hydrogen bonds with hydroxyl groups in itaconate with the distances of 2.711 Å and 2.961 Å, respectively (Fig. 4c). As shown in Fig. 4d, itaconate interacted hydrophobically with the lipophilic pocket of KEAP1- Kelch through the Arg565, Val594, Ile566, Tyr584, Tyr567, Asp585, Thr590, Trp591, and Ser592. These interaction modes may substantially increase the binding affinity between itaconate and the Kelch domain. Derived from itaconate, 4-OI plays the same role in modulating inflammation.^26,31^ It is reported that itaconate provokes Nrf2 cascade via alkylation of KEAP1 cysteine residues,^26^ and our molecular docking also indicated that itaconate could covalently bind to Cys151 in KEAP1-BTB domain, which led to the destabilization of KEAP1 dimer. Meanwhile, the cysteine residue Cys583 in Kelch domain presumably could also be alkylated by itaconate molecule. Therefore, itaconate could target KEAP1 by selectively covalently binding to conserved cysteine residues Cys151 or Cys583, subsequently disrupting the KEAP1-Nrf2 interaction. Similarly, the Co-IP assay indicated that KEAP1 immunoprecipitated with Nrf2 in the control group, while the KEAP1-Nrf2 complex was significantly disassociated in 4-OI treated group (Fig. 4e). Following disassociation, the Nrf2 protein was accumulated in the cytoplasm. Decreased KEAP1 protein expression was observed after LPS stimulation (Fig. 4f).

**Table 1:** The docking scores of cysteine residues with itaconate

Domain	BTB	Kelch				
Cysteine residues	Cys151	Cys583	Cys513	Cys489	Cys434	Cys368
Docking score (kcal/mol)	72	83	50	44	43	15

**Fig. 5:**
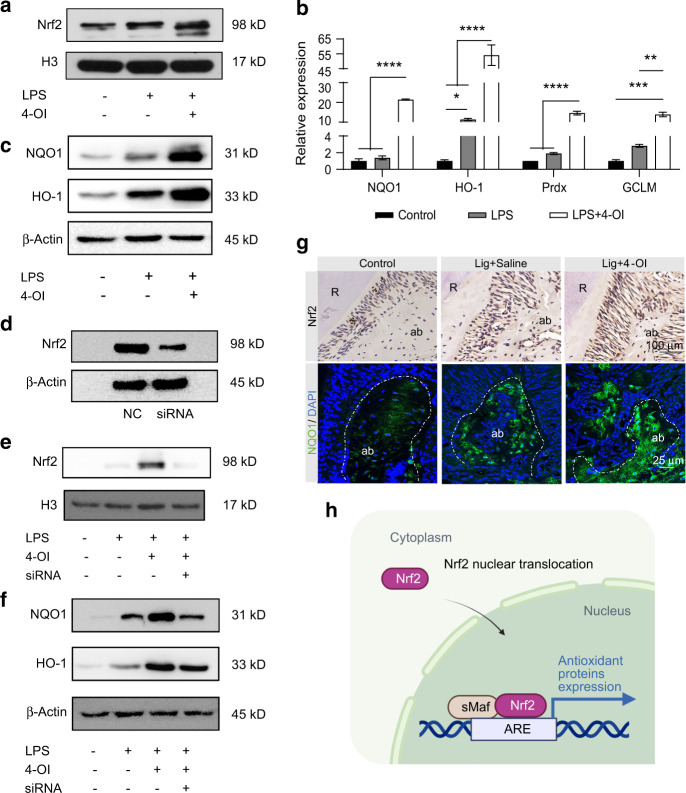
The role of Nrf2 exerted in the protection of 4-OI against periodontal inflammation. **a** The Nrf2 protein expression in nuclei fraction lysates quantified by western blotting. **b** qRT-PCR analysis of NQO1, HO-1, Prdx and GCLM in RAW264.7. **c** The expression of Nrf2 downstream ARE-dependent proteins in RAW264.7 measured by western blotting. **d** Transfection effects of siRNA or negative control siRNA (NC) analyzed by western blotting. Nrf2 expression in nuclei fraction lysates (**e**) and downstream proteins in cytosol fraction lysates (**f**) after siRNA transfection as detected by western blotting. **g** Representative images of histological analysis in periodontal tissue. (Scale bar = 100 or 25 µm). **h** Schematic diagram of the nuclear translocation of Nrf2. Data are expressed as the mean ± SD. *P < 0.05, **P < 0.01, ***P < 0.001, ****P < 0.000 1. sMaf small musculoaponeurotic fibrosarcoma

As Nrf2 protein levels were markedly upregulated in the nuclei fraction lysates of the LPS + 4-OI group, suggesting that Nrf2 protein has undergone nuclear translocation (Figs. 5a, S9a). Similarly, with the treatment of 4-OI, more overt intracellular nuclear translocation of Nrf2 was observed in IF staining (Fig. S10). Notably, with the combination of anti-inflammatory and antioxidant effects, the expression of Nrf2 downstream ARE-dependent genes in RAW264.7 and BMDMs, including HO-1, Prdx1, NQO1, and GCLM, were distinctly upregulated in 4-OI treated group (Figs. 5b, S11). Protein expressions of NQO1 and HO-1 were also remarkably increased in 4-OI treated group (Figs. 5c, S9b, c, S12). The above results indicated that 4-OI caused disassociation of KEAP1-Nrf2 by selectively covalently binding to conserved cysteine residues of KEAP1, and consequently activating Nrf2 pathway in the inflammatory periodontal microenvironment (Figs. 4g, 5g).

### Inhibition of Nrf2 impairs the 4-OI mediated antioxidant protective effect and the therapeutic effect on periodontitis

**Fig. 6:**
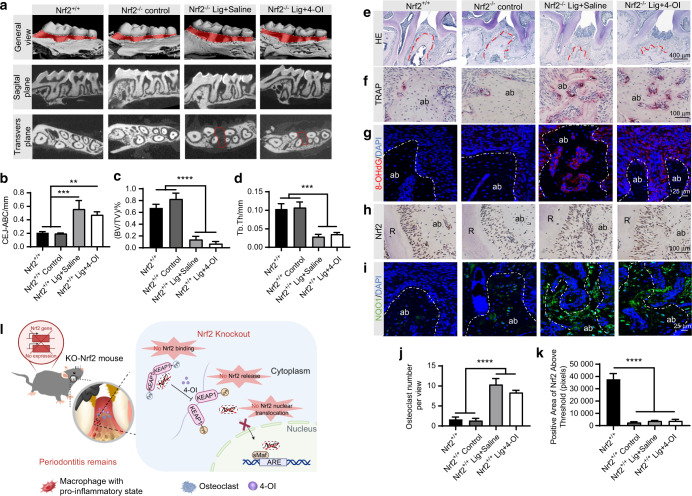
Inhibition of Nrf2 impairs the 4-OI mediated antioxidant protective effect and the therapeutic effect on inflammatory periodontal microenvironment. **a** Micro-CT image of the alveolar bone. The red part displayed the exposure of root. CEJ-ABC distance (**b**), BV/TV (**c**), and Tb. Th (**d**). **e–i** Representative images of histological analysis in periodontal tissue. (Scale bar = 400, 100, or 25 µm). **j, k** The semi-quantitative analysis of TRAP staining and IHC staining of Nrf2. **l** Illustration of local injection of 4-OI in experimental periodontitis Nrf2^−/−^ mice for 14 days. Data are presented as the mean ± SD. *P < 0.05, **P < 0.01, ***P < 0.001, ****P < 0.000 1. Lig ligature, ab alveolar bone, R root, sMaf small musculoaponeurotic fibrosarcoma

To elucidate the specific role of Nrf2 underlying the protective effect of 4-OI on periodontitis, we conducted the siRNA knockdown assay. Transfection efficiency was confirmed by qRT-PCR (Fig. S13a) and western blotting (Figs. 5d, S13b). After exposure to LPS, nuclear Nrf2 accumulation was increased compared with the control group and further substantially upregulated by 4-OI treatment. Nevertheless, a prominent decrease in the accumulation of nuclear Nrf2 was observed in the silenced group (Fig. 5e). As expected, protein expressions of NQO1 and HO-1 were congruent with the above results (Figs. 5f, S13c, d), confirming that 4-OI prevents host cells from oxidative damage by activating the Nrf2 signaling cascade. The same trend was also observed in the experimental periodontitis model in vivo (Figs. 5g, S14). Furthermore, we verified the mechanism of 4-OI in experimental periodontitis Nrf2^−/−^ mice. Compared with Nrf2^+/+^ (WT) mice, alveolar bone resorption was still evident in ligature-treated Nrf2^−/−^ mice, where 4-OI did not rescue ligature-induced bone loss (Fig. 6a–d). The same trends were observed in H&E and tartrateresistant acid phosphatase (TRAP) staining, and 4-OI treatment did not mitigate the resorption and downregulate osteoclastogenesis in the Nrf2^−/−^ ligature + 4-OI group (Fig. 6e, f, j). Additionally, elevated accumulation of 8-OHdG in Nrf2^−/−^ mice indicated the ligature noticeably caused oxidative damage, compared to the Nrf2^−/−^ control group and the Nrf2^+/+^ group. However, 4-OI treatment failed to circumvent the oxidative damage in Nrf2^−/−^ mice (Figs. 6g, S15a, b). Meanwhile, bare Nrf2 expression was exhibited in spite of 4-OI treatment or experimental induction of periodontitis in Nrf2^−/−^ mice (Figs. 6h, k, S15a). As shown in Fig. 6i, the fluorescence intensity of NQO1 was markedly suppressed in Nrf2^−/−^ mice, and 4-OI did not reverse this attenuation (Fig. S15a, c). Therefore, the experiments in vitro and in vivo confirmed that 4-OI ameliorated oxidative stress damage and alveolar bone destruction in experimental periodontitis through alkylating KEAP1’s cysteine residues to cause Nrf2-KEAP1 disassociation and consequent activation of Nrf2 pathway (Fig. 6l).

## Discussion

Our present study substantiated for the first time that 4-OI ameliorates periodontal destruction in experimental periodontitis. We validate that 4-OI activates the Nrf2 signaling cascade by alkylating the cysteine residues of KEAP1, thereby reducing periodontal destruction. In addition, it is believed that reducing robust oxidative stress may provide a new therapeutic perspective for periodontal treatment.

Given the important role of macrophages in regulating microenvironmental homeostasis, the innate immune response is considered to be a pivotal target for modulation.^32^ Transformation of macrophage phenotype in the periodontal microenvironment serves as a bridge between the pro-inflammatory and anti-inflammatory states.^33,34^ Therein, metabolic products are undoubtedly involved in this phenotype transition. Earlier this year, Trauelsen et. al. noted that succinate functioned as a classical tricarboxylic acid (TCA) cycle metabolite and efficiently regulated the transcription of immune function genes in macrophages.^35^ Another study illustrated that arachidonic acid inhibited the polarization of macrophages to the M2 type, but its derived metabolite prostaglandin E2 (PGE2)^36^ inversely facilitated the M2 polarization. Thus, metabolites in the tissue microenvironment can modulate the polarization of macrophages, which could lead to distinct outcomes in various diseases.

Itaconate, an endogenous metabolite diverted from the TCA cycle, has been proven to possess a prominent immune-regulated function.^37^ In macrophages, numerous stimuli (such as LPS) can induce the production of itaconate (Fig. 2b). However, the stimuli also lead to the accumulation of the pro-inflammatory M1 phenotype, which is highly implicated in tissue damage.^38^ Consistent with our results, despite the presence of itaconate, it was not sufficient to counteract the excessive inflammatory response induced by LPS (Fig. 2c), suggesting that exogenous supplementation of metabolite should be required to reverse the oxidative damage and modulate the local inflammatory state.

4-OI is a cell-permeable itaconate derivative, making it a suitable itaconate surrogate. Although the 4-OI has been applied to systematic diseases,^26,39,40^ the critical role of 4-OI-exerting protective effects on inflammation control and alveolar bone loss in periodontitis has been discussed in our study for the first time (Fig. 1). Since 4-OI had similar thiol reactivity to itaconate, it could alkylate cysteine residues of KEAP1,^26^ and allow newly released Nrf2 to translocate to the nucleus and subsequently activate Nrf2 cascade.^27^ In our study, 4-OI performed the anti-inflammatory function by suppressing the M1-phenotype polarization and promoting the phenotypic switch to M2 (Fig. 2d, e). In addition, 4-OI induced a significantly decreased cytokines levels (Fig. 2f–h), and consequently ameliorated the inflammation in the periodontal microenvironment. Moreover, consistent with the related researches,^27,41,42^ our results also indicated that 4-OI could effectively scavenge excess ROS and minimize oxidative damage in the inflammatory periodontal microenvironment; thereby re-confirming the protective effect of 4-OI against ROS.

Nrf2 serves as a master regulator of cellular defenses against oxidative stress that activates antioxidant and detoxifying enzymes. Therapeutic strategy to activate Nrf2 signaling pathway can effectively protect macrophages from oxidative damage.^17,43^ Our results confirmed that the activation of Nrf2 pathway was required for 4-OI-induced protection of host cells against oxidative stress.^27,42^ This protection was accomplished via activation of Nrf2 signaling cascade by 4-OI that led to KEAP1-Nrf2 disassociation and nuclear translocation (Figs. 5a, S10). In addition, 4-OI treatment upregulated ARE activity as well as mRNA and protein expression. Moreover, knockdowning Nrf2 markedly suppressed antioxidant capacity in macrophages in spite of 4-OI treatment in vitro (Fig. 5f). Notably, 4-OI failed to ameliorate ligature-induced oxidative damage and alveolar bone resorption in Nrf2^−/−^ mice models (Fig. 6). The above results clearly elucidated that 4-OI attenuates periodontal inflammatory damage through activation of Nrf2-mediated antioxidant defense mechanisms. However, multiple clinical trials with larger sample sizes and longer timepoints are needed to validate the effectiveness of 4-OI.

In summary, as an activator of Nrf2, 4-OI was first discovered to inhibit periodontal destruction by activating Nrf2 signaling cascade. Due to its superior anti-inflammatory, antioxidant, and osteoclastogenesis inhibiting abilities, 4-OI is expected to be a promising candidate in periodontal topical treatment.

## Materials and methods

### Mice experimental periodontitis models

All animal experimental procedures in this study were approved by the Ethics Committee of Chongqing Medical University. In mice modeling, we applied the silk sutures with both ends tied knots to insert between the maxillary first molar and second molar. The check of ligation was done on the daily basis in case of loosening or displacement. The contralateral side of maxillae without ligature treatment as a control. The ligature was retained for 2 weeks in order to establish experimental periodontitis.^44^ Animals were fed with nutrients and distilled water ad libitum at the Chongqing Key Laboratory of Oral Diseases and Biomedical Sciences.

### Animal group allocation

Six-week-old C57BL/6 male mice were randomly divided into three groups (*n* = 10 per group)^45^: (1) Control group (no treatment), (2) Lig + Saline group (ligature-induced experimental periodontitis and saline injection for 14 days), and (3) Lig + 4-OI group (experimental periodontitis and 4-OI injection for 14 days). In another part, six-week-old Nrf2^−/−^ and WT C57BL/6 male mice were divided into four groups (*n* = 10 per group): (1) Nrf2^+/+^ group (WT with no treatment), (2) Nrf2^−/−^ Control group (Nrf2^−/−^ with no treatment), (3) Nrf2^−/−^ Lig + Saline group (Nrf2^−/−^ with ligature-induced experimental periodontitis and saline injection for 14 days), (4) Nrf2^−/−^ Lig + 4-OI group (Nrf2^−/−^ with experimental periodontitis and 4-OI injection for 14 days). One microliter of 4-OI (50 mg/kg) was daily injected into the gingival tissue at the interdental site between the maxillary first molar and second molar, and the other group was simultaneously injected with saline (1 μL) at the same site for 14 days as a control.

### Radiographic analysis

Animals were sacrificed by CO_2_ hypoxia after 14 days post-operation. The maxillae were separated and scanned by micro-CT (vivaCT80, SCANCO Medical AG, Switzerland). The scanning parameters were set at 70 kV and 112 μA, with a thickness of 10 μm per slice. The region of interest (ROI) was a cuboidal bone body encompassing the alveolar bone. The ROI length extended from the most distal aspect of the upper first molar (M1) root to the most mesial aspect and upper second molar (M2) root. Its width extended from the most buccal to the most palatal aspect of the M1 or M2 roots, and the height extended from the most apical aspect of M1 or M2 root to the most coronal part of the alveolar bone crest (ABC). And the cementoenamel junction and alveolar bone crest (CEJ-ABC) distance, bone volume per tissue volume (BV/TV) percentage, and trabecular thickness (Tb.Th) of ROI were analyzed by using the SCANCO VivaCT40 micro-CT software.

### Histological analyses

All samples were fixed in 4% PFA solution for 1 day and demineralized in 17% ethylenediaminetetraacetic acid for 28 days. The maxillae specimens were subsequently embedded in paraffin and sections of 6 μm thickness were prepared. Alveolar bone loss was evaluated by hematoxylin and eosin (H&E) staining and osteoclast activity was examined by tartrateresistant acid phosphatase (TRAP) staining. Osteoclasts were identified by both TRAP-positive signs and nuclei of more than 3. TRAP staining was analyzed by three blinded observers and quantified on the bone defect from three TRAP-stained sections per group 14 days after surgery with the Olympus BX41 microscope.

### Immunostaining and histomorphometric analyses

Immunostaining followed published protocols.^46^ In short, Tissue sections were de-paraffinized following standard procedures and incubated overnight with primary antibodies against F4/80 (1:200, Abcam, USA), iNOS (1:500, Abcam, USA), CD206 (1:800, Abcam, USA), IL-6 (1:500, Abcam, USA), Nrf2 (1:200, Bioss, China) and NQO1(1:200, Abcam, USA) at 4 °C. On the next day, sections for IF staining were incubated with the corresponding fluorescently labeled secondary antibody and DAPI (staining nucleus), and sections for IHC staining were incubated with the corresponding secondary antibody and counterstained with hematoxylin (staining nucleus). For the semi-quantification analysis of IHC part, images were analyzed in ImageJ software (NIH, Bethesda, USA) using the “IHC Toolbox” plugin. Three blinded examiners performed the semi-quantification analysis, and at least three consecutive slides of each sample were used. And for the semi-quantification analysis of IF part, 3D surface plot in Image J (NIH, Bethesda, USA) was performed, the higher peak indicated more robust infiltration.

### Cell culture

Mouse macrophage cell line RAW264.7 was purchased from the Chinese Academy of Typical Culture Collection Cell Bank (Shanghai, China). BMDMs were isolated from the leg bones of C57BL/6J mice and differentiated in Dulbecco’s modified Eagle’s complete medium supplemented with 25 ng/mL M-CSF for 6 days. RAW264.7 cells and BMDMs were maintained in DMEM (Gibco, USA), supplemented with 10% fetal bovine serum (Gibco, USA) and penicillin-streptomycin solution at 37 °C with 5% CO_2_. Human periodontal ligament cells (hPDLCs) were isolated and cultured according to previously published protocol.^47^ hPDLCs at two to four passages (P2–P4) were used for further treatment.

### Cell treatment

For the purpose of establishing the inflammatory and oxidative stress model in vitro, RAW264.7 cells and BMDMs were exposed to the *Porphyromonas. gingivalis* LPS (100 ng·mL^−1^ and 10 ng·mL^−1^, respectively) (Sigma) for 24 h after reaching ~70% confluence. Purchased from MedChemExpress (MonmouthJunction, NJ, USA), 4-OI was dissolved in DMSO and then stored at −80 °C. It was subsequently diluted to ensure that the DMSO concentration was 0.1%. Equal amounts of DMSO were added to the control group. The pre-treatments of 4-OI occurred before LPS stimulation was for 3 h.

### Ultrahigh performance liquid chromatography mass spectroscopy (UPLC-MS)

We utilized UPLC-MS to investigate the intracellular concentration of itaconate. The details were described in the previously published article.^40^ Briefly, the 6 cm dishes were used for macrophages seeding at a density of 6 × 10^5^ cells per dish overnight. After 24 h LPS stimulation, samples were collected in tubes and next quenched with liquid nitrogen. Following the addition of extraction solution, samples were centrifuged at 12 000 r·min^−1^ for 10 min and the supernatant was then transferred for further analysis. Liquid chromatographic separation of the samples was conducted using an Agilent 1290 Infinity II series ultra-performance liquid chromatography system (Agilent Technologies, Santa Clara, CA, USA). Meanwhile, Agilent MassHunter workstation software (B.08.00; Agilent Technologies) was used for data processing.

### Cell viability

A CCK-8 assay was utilized to determine the proper concentration of 4-OI. PDLCs were seeded into 96-well plates at a density of 4 × 10^3^ cells per well and incubated overnight, and then the culture media were changed and supplemented with various concentrations of 4-OI. After incubation for 1, 4, and 7 days, 10 μL of CCK-8 solution was subsequently added to each well for 2 h treatment. The optical density (OD) was examined at 450 nm using a multimode plate reader (PerkinElmer).

### Quantitative real-time PCR (qRT-PCR)

The total cellular RNA was harvested by Trizol reagent (Thermo Fisher Scientific), and used for reverse transcription. qRT-PCR was next performed on a ProFlex PCR system (Thermo Fisher, USA) using TB green PCR Master Mix (Takara). Glyceraldehyde-3-phosphate dehydrogenase (GAPDH) was adopted as the reference gene. And the 2^−ΔΔCt^ method was used to calculate the target gene expression. The primer sequences for genes are listed in Table S1.

### Immunofluorescence staining

After treatment, samples were fixed in 4% paraformaldehyde for 15 min and permeabilized with 0.1% Triton X-100 for 20 min. Next, samples were incubated with anti-iNOS (1:500, Abcam, USA), anti-CD86 (1:100, Abcam, USA), anti-CD206 (1:800, Abcam, USA), anti-8-Hydroxy-2’-deoxyguanosine (8-OHdG) (1:200, Bioss, China) and anti-Nrf2 (1:200, Abcam, USA), followed by incubation with the corresponding secondary antibody and DAPI. The immunofluorescence images were captured by LSCM. 3D surface plot was analyzed by Image J (NIH, Bethesda, USA). The higher the peak indicated more robust infiltration.

### Western blotting

Extraction of cell proteins was accomplished by applying RIPA lysis buffer (Beyotime, China), and the total protein concentration was measured by the Enhanced BCA Protein Assay Kit (Beyotime, China). The proteins were separated by SDS-PAGE and then transferred onto polyvinylidene fluoride (PVDF) membranes (Millipore, USA). After being blocked in 5% skim milk solution, the membranes were incubated with primary antibodies overnight at 4 °C. The bands were incubated with antibodies specific for CAT (1:1 000, Abcam, USA), Nrf2 (1:1 000, CST, USA), KEAP1 (1:1 000, CST, USA), NQO1(1:1 000, Abcam, USA) and HO-1 (1:4 000, Abcam, USA) overnight at 4 °C. Histone H3 (1:3 000, Abcam, USA), β-Actin (1:1 000, ZenBio, China) and GAPDH (1:1 000, ZenBio, China) were used as the internal control. The PVDF membranes were incubated with secondary antibodies at room temperature. Subsequently, the chemiluminescent reagent (Merck Millipore, USA) and imaging system (LAS4000M, USA) were used to visualize the target protein bands. Finally, the bands were quantified by ImageJ software (NIH, USA).

### Enzyme-linked immunosorbent assay (ELISA)

To assess the concentrations of IL-6 in each group, the supernatant was collected and assayed using ELISA kits (DVE00; R&D Systems) according to the manufacturers’ instructions. The OD value was measured at the wavelength of 450 nm, and the concentrations of IL-6 were calculated based on a standard curve. Results are expressed as pg·mL^−1^.

### Profiling of the immune microenvironment by Mouse Cytokine Array Kit

The mouse proteome profiler cytokines array kit (R&D System, USA), which contained 40 unique cytokines, was used to analyze inflammatory states in different groups. The supernatant was collected and analyzed according to the instructions. In the data readout, every two adjacent dots on the membrane represent one kind of cytokine. The representative upregulation (up, cytokines of the LPS group compared to the control group) and downregulation (down, cytokines of the LPS + 4-OI group compared to the LPS group) were marked in red and blue boxes, respectively. Besides, the expression profile of cytokines with significant differences was depicted by a radar chart.

### Detection of oxidative stress

Cells were plated in six-well plates with a density of 10^5^ per well for further detection. The intracellular ROS level was measured by ROS Assay Kit (Beyotime, China). The fluorescence intensity of DCFH-DA was read under a microplate reader and a BD Influx flow cytometer (BD Biosciences, USA), using excitation and emission wavelengths of 488 and 525 nm. The fluorescence images were observed by fluorescence microscopy and data was analyzed using FlowJo software. Lipid peroxidation levels reflect the extent of intracellular oxidative damage, as measured by Lipid Peroxidation MDA Assay Kit (Beyotime, China). Besides, the Total Superoxide Dismutase Assay Kit with WST-8 method was applied to test the activity of superoxide dismutase (SOD).

### Molecular docking simulation

The geometry of itaconate (Compound PubChem CID: 811) and the crystal structures of KEAP1 (PDB ID: 5DAF, 2FLU) were downloaded from the National Center for Biotechnology Information (http://pubchem.ncbi.nlm.nih.gov) and Protein Data Bank, respectively. Water molecules were removed, and hydrogen atoms were then added to the crystal structure. The docking for itaconate towards the BTB and Kelch domains in KEAP1 has been done using GOLD.^48^ The program optimizes the geometry for hydrogen bonding by allowing the rotation of hydroxyl and amino groups contained in the amino acid chain. The docking area of the binding site was centered on the cysteine residues in BTB and Kelch domains. Finally, using the covalent docking method, the ligands were covalently docked by Michael addition reaction. The latest best pose for itaconate was selected using the docking score. The number of genetic operations (crossing, migration, mutation) during the search procedure was set as automatic. According to the library, the side-chain rotamers were defined. Previous studies indicated that ChemPLP is the best scoring function in molecular docking studies, which is one of the functions of the GOLD program (ChemPLP, GoldScore, ChemScore, or ASP). The score of each pose identified is calculated as the negative of the sum of a series of energy terms involved in the protein-ligand interaction process, so that the more positive the score, the better is the interaction. For viewing the docked projects and generating images, UCSF Chimera candidate was used.^49^

### Co-immunoprecipitation (Co-IP)

Co-IP assay was performed as previously described in Zheng et al.^27^ Whole-cell lysates were centrifuged at 12 000 r·min^−1^ for 15 min at 4 °C and then took the supernatant as total protein lysates. BCA method was carried out to measure the protein concentration. Pre-chilled TBS was used to wash Pierce^TM^ Protein A/G Agarose Beads (Thermo Fisher Scientific, USA) twice and the beads were then transferred into 50% suspension with diluent. To remove non-specific adsorption, the lysates were pre-washed with Protein A/G agarose, and centrifuged at 2 500 r·min^−1^ for 3 min at 4 °C to take the supernatant. In order to avoid the consumption of antigen by free antibody, the anti-KEAP1 antibody and Protein A/G agarose were pre-incubated. Then, the pretreated beads were added to the lysates and incubated with anti-KEAP1 antibody overnight at 4 °C, and centrifuged at 2 500 r·min^−1^ for 3 min. After that, we added 100 μL of 2 × SDS loading buffer to the samples and incubated them at 95 °C for 5 min, and the proteins were consequently subjected to western blotting analysis.

### Transfection

The protocol was performed according to the previous study.^26^ On the day of transfection, two Eppendorfs were prepared. Opti-MEM medium (250 μL per well) was added to each tube. The RNAimax reagent was diluted in one set of tubes and siRNA of Nrf2 in the second set of tubes. Then, we mixed the liquids in the two tubes in equal proportions and incubated them at room temperature. The mix was added to each well. After 24 h transfection, cells were treated as required. Nrf2 siRNA sequences are presented in Table S2.

### Statistical analysis

All results were shown as the means ± standard deviations (SD). The Student’s *t*-test and one-way analysis of variance (ANOVA) were used to determine the statistical significance. GraphPad Prism 8.0 (San Diego, USA) software was then used for all statistical analyses, and statistical significance was considered at *P* < 0.05. All experiments were performed at least in triplicate and repeated three times.

## Supplementary information

Four-Octyl itaconate ameliorates periodontal destruction via Nrf2-dependent antioxidant system

## Data Availability

All data associated with this study are presented in the paper.

## References

[1] Kinane DF, Stathopoulou PG, Papapanou PN (2017). Periodontal diseases. Nat. Rev. Dis. Prim..

[2] Pihlstrom BL, Michalowicz BS, Johnson NW (2005). Periodontal diseases. Lancet (Lond., Engl.).

[3] Yamada H, Nakajima T, Domon H, Honda T, Yamazaki K (2015). Endoplasmic reticulum stress response and bone loss in experimental periodontitis in mice. J. Periodontal Res..

[4] Allen EM, Matthews JB, DJ OH, Griffiths HR, Chapple IL (2011). Oxidative and inflammatory status in Type 2 diabetes patients with periodontitis. J. Clin. Periodontol..

[5] Hajishengallis G, Chavakis T (2021). Local and systemic mechanisms linking periodontal disease and inflammatory comorbidities. Nat. Rev. Immunol..

[6] Li X, Kolltveit KM, Tronstad L, Olsen I (2000). Systemic diseases caused by oral infection. Clin. Microbiol. Rev..

[7] Schenkein HA, Papapanou PN, Genco R, Sanz M (2020). Mechanisms underlying the association between periodontitis and atherosclerotic disease. Periodontology.

[8] Choi SE, Sima C, Pandya A (2020). Impact of treating oral disease on preventing vascular diseases: a model-based cost-effectiveness analysis of periodontal treatment among patients with type 2 diabetes. Diabetes Care.

[9] Hajishengallis G, Lambris JD (2013). Complement-targeted therapeutics in periodontitis. Adv. Exp. Med. Biol..

[10] Bao X, Zhao J, Sun J, Hu M, Yang X (2018). Polydopamine nanoparticles as efficient scavengers for reactive oxygen species in periodontal disease. ACS Nano.

[11] Chen FM, Jin Y (2010). Periodontal tissue engineering and regeneration: current approaches and expanding opportunities. Tissue Eng. Part B Rev..

[12] Nabet BY (2017). Exosome RNA unshielding couples stromal activation to pattern recognition receptor signaling in cancer. Cell.

[13] Takeuchi O, Akira S (2010). Pattern recognition receptors and inflammation. Cell.

[14] Van den Bossche J (2016). Mitochondrial dysfunction prevents repolarization of inflammatory macrophages. Cell Rep..

[15] Saravanakumar G, Kim J, Kim WJ (2017). Reactive-oxygen-species-responsive drug delivery systems: promises and challenges. Adv. Sci..

[16] Sczepanik FSC (2020). Periodontitis is an inflammatory disease of oxidative stress: we should treat it that way. Periodontology.

[17] Chiu AV, Saigh MA, McCulloch CA, Glogauer M (2017). The role of NrF2 in the regulation of periodontal health and disease. J. Dent. Res..

[18] Wang GP (2015). Defining functional signatures of dysbiosis in periodontitis progression. Genome Med..

[19] Hirschfeld, J., White, P. C., Milward, M. R., Cooper, P. R. & Chapple, I. L. C. Modulation of neutrophil extracellular trap and reactive oxygen species release by periodontal bacteria. *Infection immunity* **85**, e00297-17 (2017). 10.1128/IAI.00297-17 PMC5695129 28947649

[20] Mjaavatten MD, Bykerk VP (2013). Early rheumatoid arthritis: the performance of the 2010 ACR/EULAR criteria for diagnosing RA. Best. Pract. Res. Clin. Rheumatol..

[21] Sfyroeras GS, Roussas N, Saleptsis VG, Argyriou C, Giannoukas AD (2012). Association between periodontal disease and stroke. J. Vasc. Surg..

[22] Fine N (2016). Distinct oral neutrophil subsets define health and periodontal disease states. J. Dent. Res..

[23] Chen M (2019). Oxidative stress-related biomarkers in saliva and gingival crevicular fluid associated with chronic periodontitis: A systematic review and meta-analysis. J. Clin. Periodontol..

[24] Kaspar JW, Niture SK, Jaiswal AK (2009). Nrf2:INrf2 (Keap1) signaling in oxidative stress. Free Radic. Biol. Med..

[25] Sima C (2016). Nuclear factor erythroid 2-related factor 2 down-regulation in oral neutrophils is associated with periodontal oxidative damage and severe chronic periodontitis. Am. J. Pathol..

[26] Mills EL (2018). Itaconate is an anti-inflammatory metabolite that activates Nrf2 via alkylation of KEAP1. Nature.

[27] Zheng Y (2020). Four-octyl itaconate activates Nrf2 cascade to protect osteoblasts from hydrogen peroxide-induced oxidative injury. Cell Death Dis..

[28] Xin Y, Zou L, Lang S (2021). 4-Octyl itaconate (4-OI) attenuates lipopolysaccharide-induced acute lung injury by suppressing PI3K/Akt/NF-κB signaling pathways in mice. Exp. Ther. Med..

[29] Zhuang Z (2019). Induction of M2 macrophages prevents bone loss in murine periodontitis models. J. Dent. Res..

[30] Ivashkiv LB (2013). Epigenetic regulation of macrophage polarization and function. Trends Immunol..

[31] Liao ST (2019). 4-Octyl itaconate inhibits aerobic glycolysis by targeting GAPDH to exert anti-inflammatory effects. Nat. Commun..

[32] Qiao W (2021). Sequential activation of heterogeneous macrophage phenotypes is essential for biomaterials-induced bone regeneration. Biomaterials.

[33] Andreev D (2020). Osteocyte necrosis triggers osteoclast-mediated bone loss through macrophage-inducible C-type lectin. J. Clin. Investig..

[34] Takayanagi H (2007). Osteoimmunology: shared mechanisms and crosstalk between the immune and bone systems. Nat. Rev. Immunol..

[35] Trauelsen M (2021). Extracellular succinate hyperpolarizes M2 macrophages through SUCNR1/GPR91-mediated Gq signaling. Cell Rep..

[36] Xu M (2021). Arachidonic acid metabolism controls macrophage alternative activation through regulating oxidative phosphorylation in PPARγ dependent manner. Front. Immunol..

[37] Li Y (2020). 4-Octyl Itaconate alleviates lipopolysaccharide-induced acute lung injury in mice by inhibiting oxidative stress and inflammation. Drug Des. Dev. Ther..

[38] Kato H, Taguchi Y, Tominaga K, Umeda M, Tanaka A (2014). Porphyromonas gingivalis LPS inhibits osteoblastic differentiation and promotes pro-inflammatory cytokine production in human periodontal ligament stem cells. Arch. Oral. Biol..

[39] Li R, Zhang P, Wang Y, Tao K (2020). Itaconate: a metabolite regulates inflammation response and oxidative stress. Oxid. Med. Cell. Longev..

[40] Sun X (2019). Octyl itaconate inhibits osteoclastogenesis by suppressing Hrd1 and activating Nrf2 signaling. FASEB J..

[41] Liu H (2018). Four-octyl itaconate activates Keap1-Nrf2 signaling to protect neuronal cells from hydrogen peroxide. Cell Commun. Signal..

[42] Tang C, Tan S, Zhang Y, Dong L, Xu Y (2019). Activation of Keap1-Nrf2 signaling by 4-octyl itaconate protects human umbilical vein endothelial cells from high glucose. Biochem. Biophys. Res. Commun..

[43] Kobayashi EH (2016). Nrf2 suppresses macrophage inflammatory response by blocking proinflammatory cytokine transcription. Nat. Commun..

[44] Weng Y (2021). Trem2 mediated Syk-dependent ROS amplification is essential for osteoclastogenesis in periodontitis microenvironment. Redox Biol..

[45] Wei W (2019). Activation of the STAT1 pathway accelerates periodontitis in Nos3(-/-) mice. J. Dent. Res..

[46] Xin L (2020). Histological and histomorphometric evaluation of applying a bioactive advanced platelet-rich fibrin to a perforated schneiderian membrane in a maxillary sinus elevation model. Front. Bioeng. Biotechnol..

[47] He Y (2018). CoCl(2) induces apoptosis via a ROS-dependent pathway and Drp1-mediated mitochondria fission in periodontal ligament stem cells. Am. J. Physiol. Cell Physiol..

[48] Verdonk ML, Cole JC, Hartshorn MJ, Murray CW, Taylor RD (2003). Improved protein-ligand docking using GOLD. Proteins.

[49] Pettersen EF (2004). UCSF Chimera-a visualization system for exploratory research and analysis. J. Comput. Chem..

