# Supplementary material for: Four-Octyl itaconate ameliorates periodontal destruction via Nrf2-dependent antioxidant system
Source: Int J Oral Sci. 2022 May 31;14:27. doi: 10.1038/s41368-022-00177-1 (PMC9151820; doi:10.1038/s41368-022-00177-1)
Supplement: Supplementary file 1 — Four-Octyl itaconate ameliorates periodontal destruction via Nrf2-dependent antioxidant system [file 41368_2022_177_MOESM1_ESM.doc]

**Four-Octyl itaconate inhibits periodontal destruction via Nrf2-dependent antioxidant system**

Liangjing Xin^1 #^, Fuyuan Zhou^1 #^, Chuangwei Zhang^1^, Wenjie Zhong^1^, Shihan Xu^1^, Xuan Jing^1^, Dong Wang^2^, Si Wang^1^, Tao Chen^1^*, Jinlin Song^1^*

^1^College of Stomatology, Chongqing Medical University, Chongqing Key Laboratory for Oral Diseases and Biomedical Sciences, Chongqing Municipal Key Laboratory of Oral Biomedical Engineering of Higher Education, Chongqing, 401147, P. R. China.

^2^Department of Ultrasound, The First Affiliated Hospital, Chongqing Medical University, Chongqing 400042, P.R. China

**
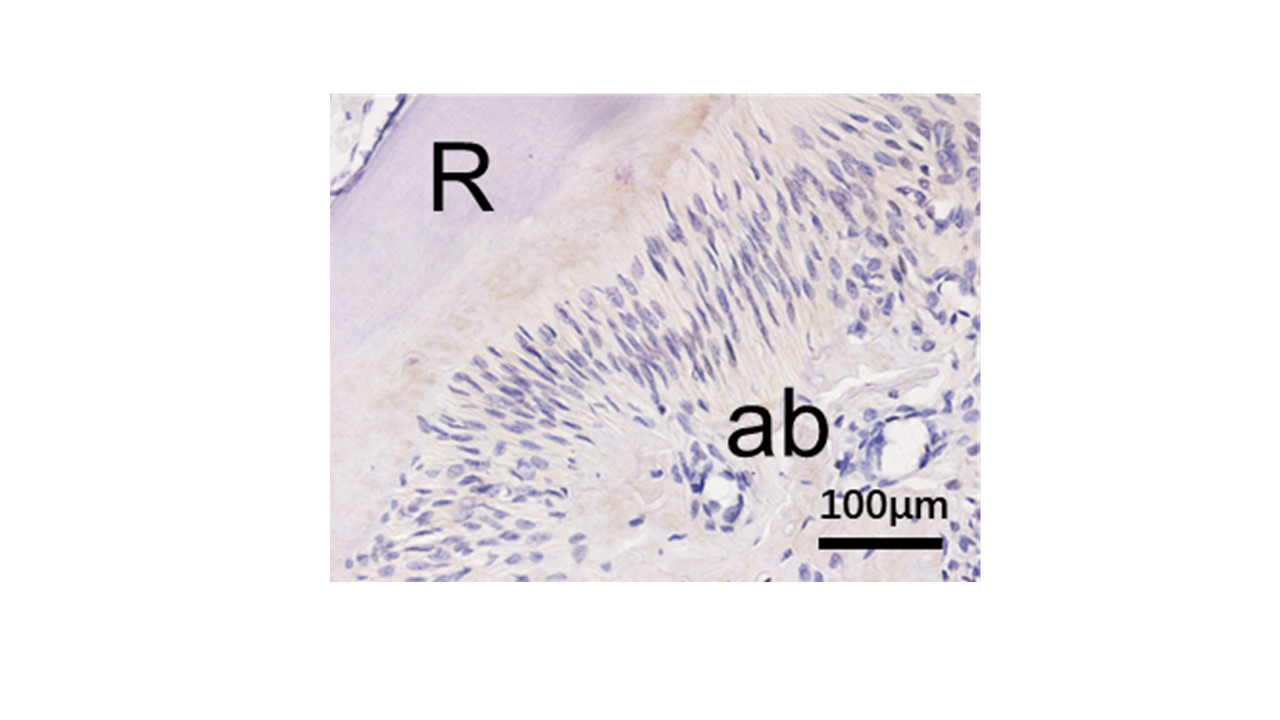
**

Figure S1: Negative control of IL-6 using IHC staining. ab: alveolar bone; R: root. (Scale bar = 100 μm)


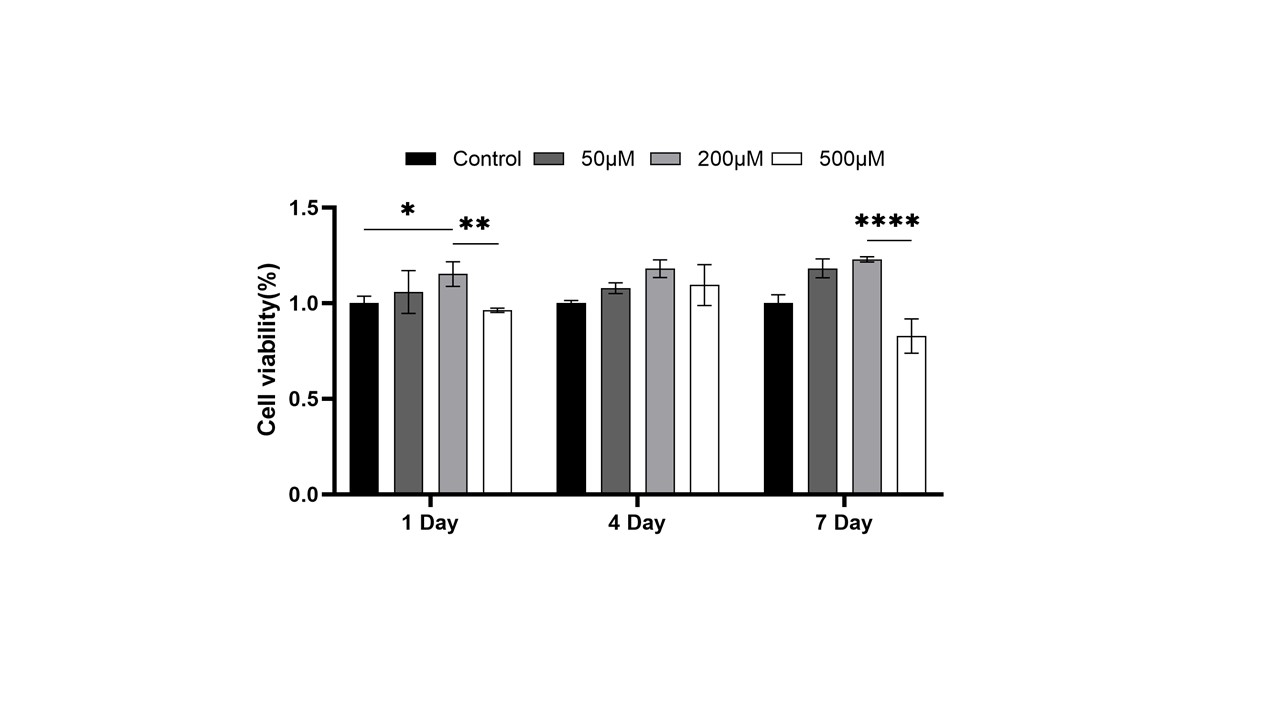


Figure S2: The viability of human periodontal ligament cells (hPDLCs) under different concentrations of 4-OI determined by CCK-8 assay.


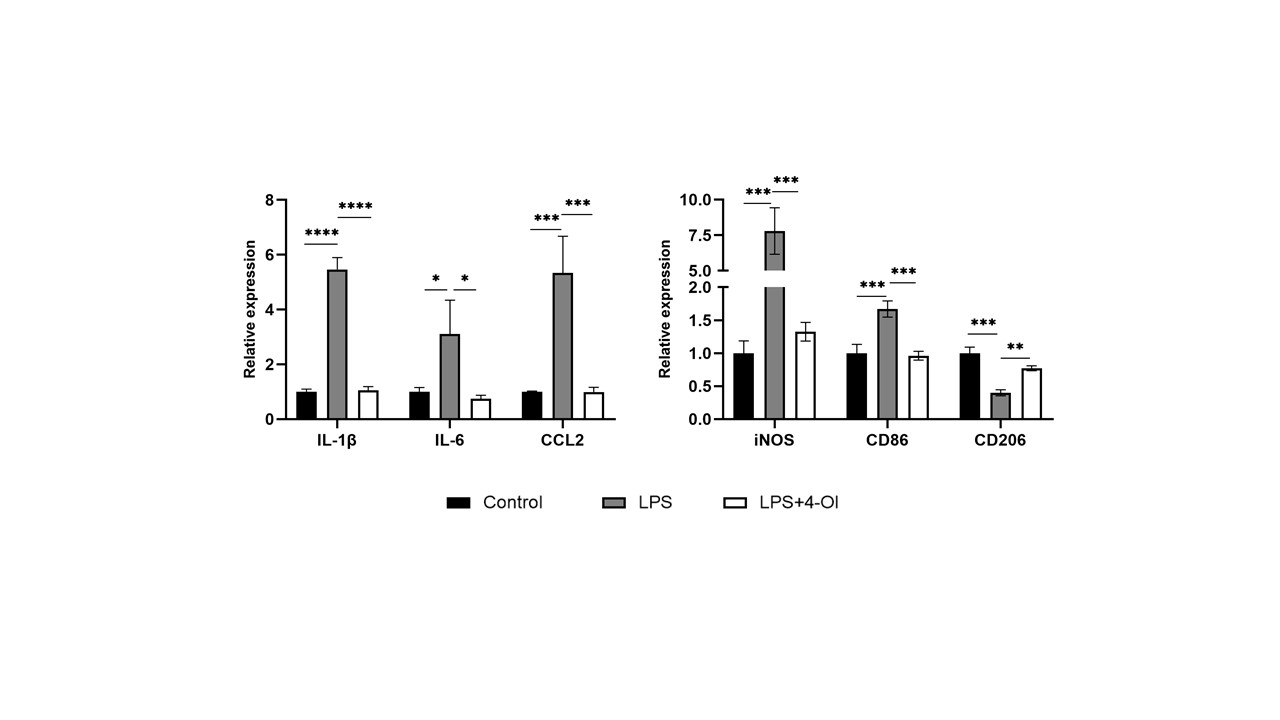


Figure S3: Expression of inflammatory genes on BMDMs using qRT-PCR.


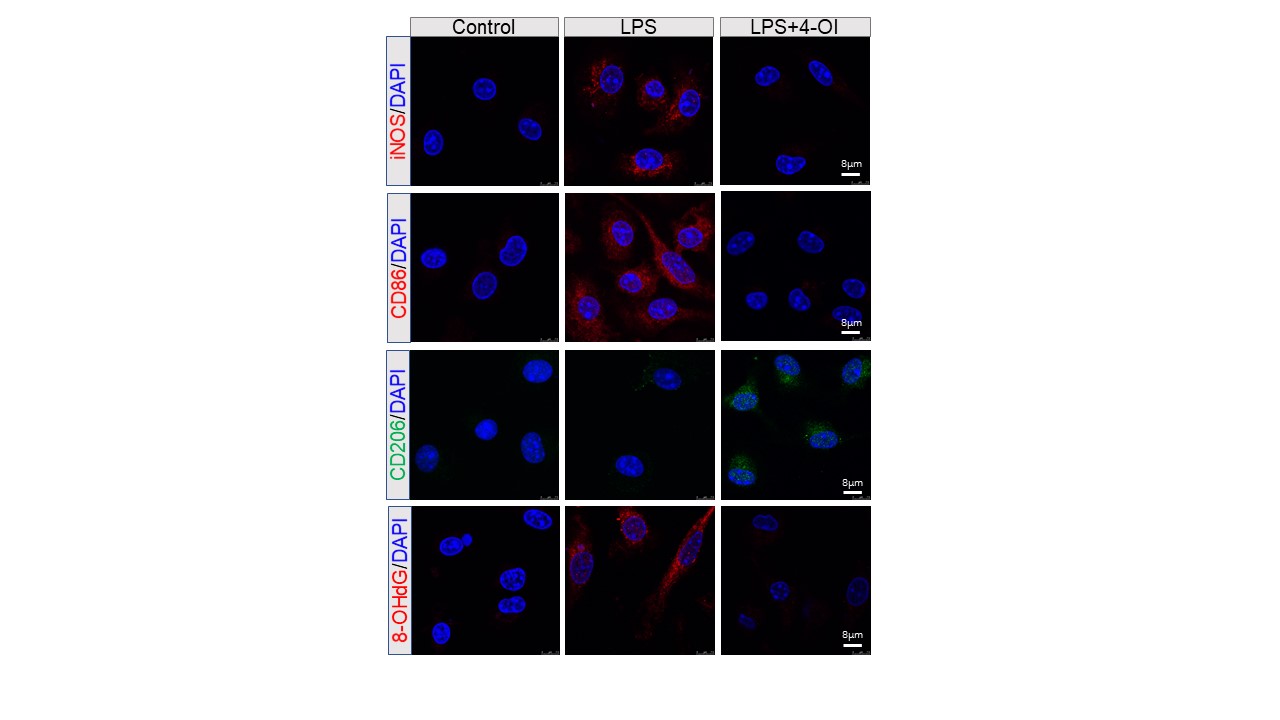


Figure S4: The IF staining of iNOS, CD86, CD206 and 8-OHdG on BMDMs using confocal laser scanning microscopy. (Nucleus: blue, 8-OHdG, iNOS and CD86: red, CD206: green. Scale bar = 8 μm)

**
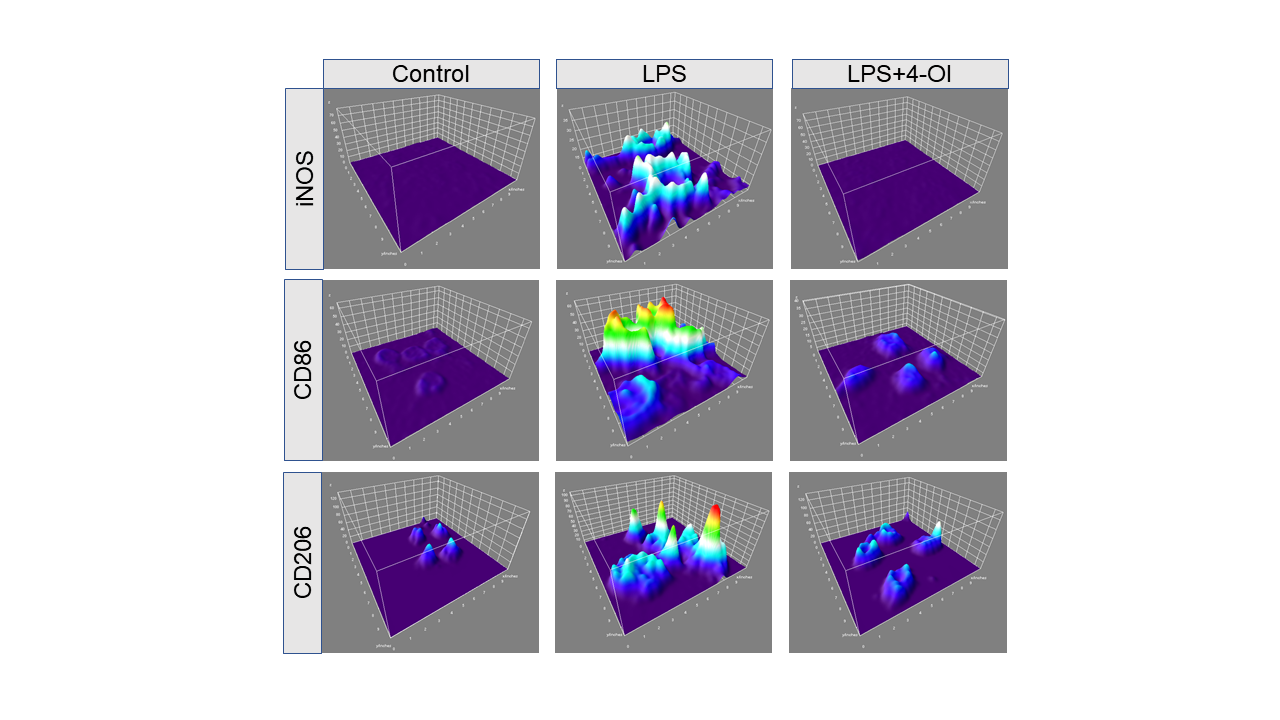
**

Figure S5: 3D surface plot images of iNOS, CD86 and CD206 on RAW264.7.


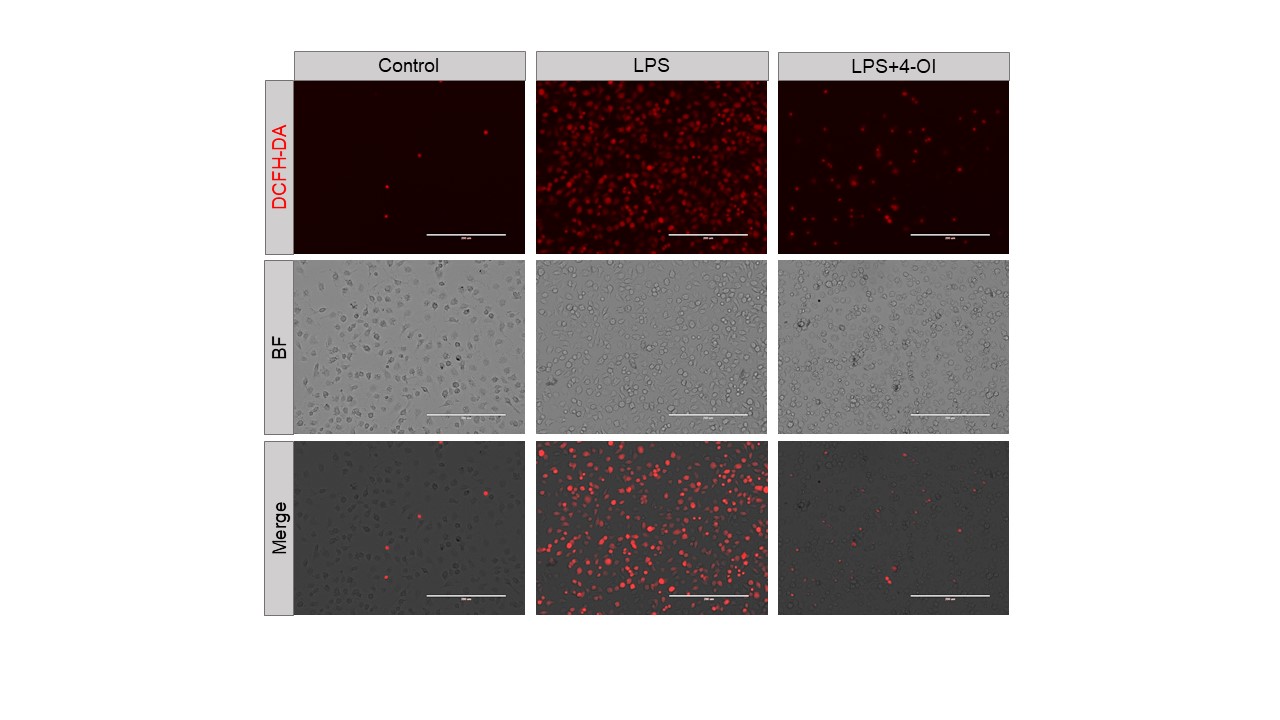


Figure S6: Fluorescent images of the intracellular ROS in BMDMs measured by DCFH-DA staining. (Scale bar = 200 µm).


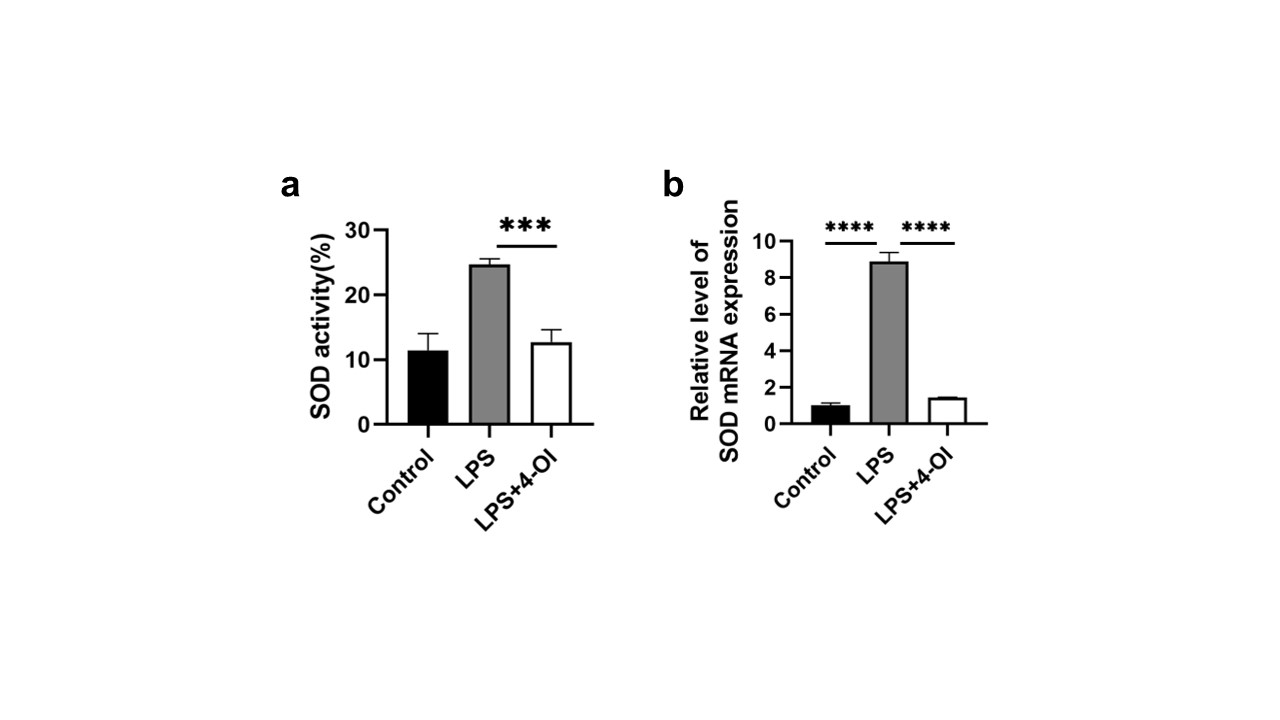


Figure S7: (a) The activity of SOD. (b) The relative level of SOD mRNA expression. Data are presented as the mean ± SD. ^***^*p*< 0.001; ^****^*p*<0.0001.


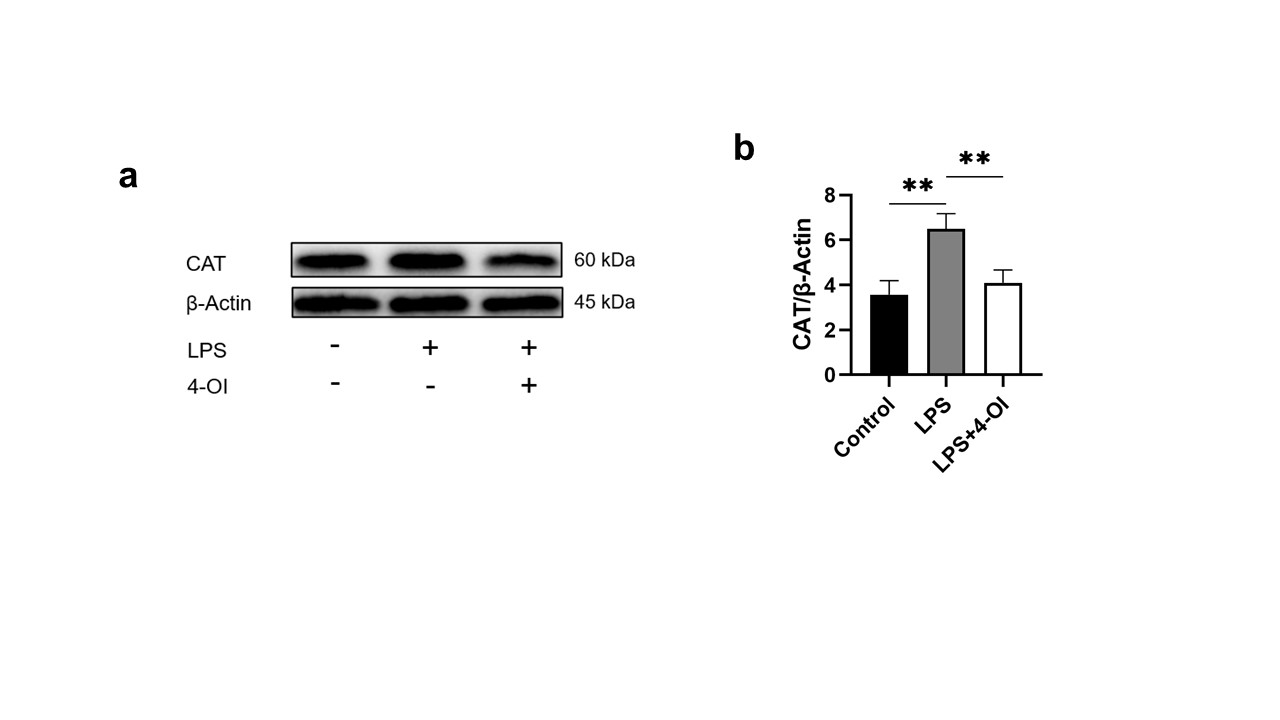
Figure S8: (a) Expression of CAT examined by western blotting. (b) The quantitative expression of CAT. Data are presented as the mean ± SD. ^**^*p*<0.01.


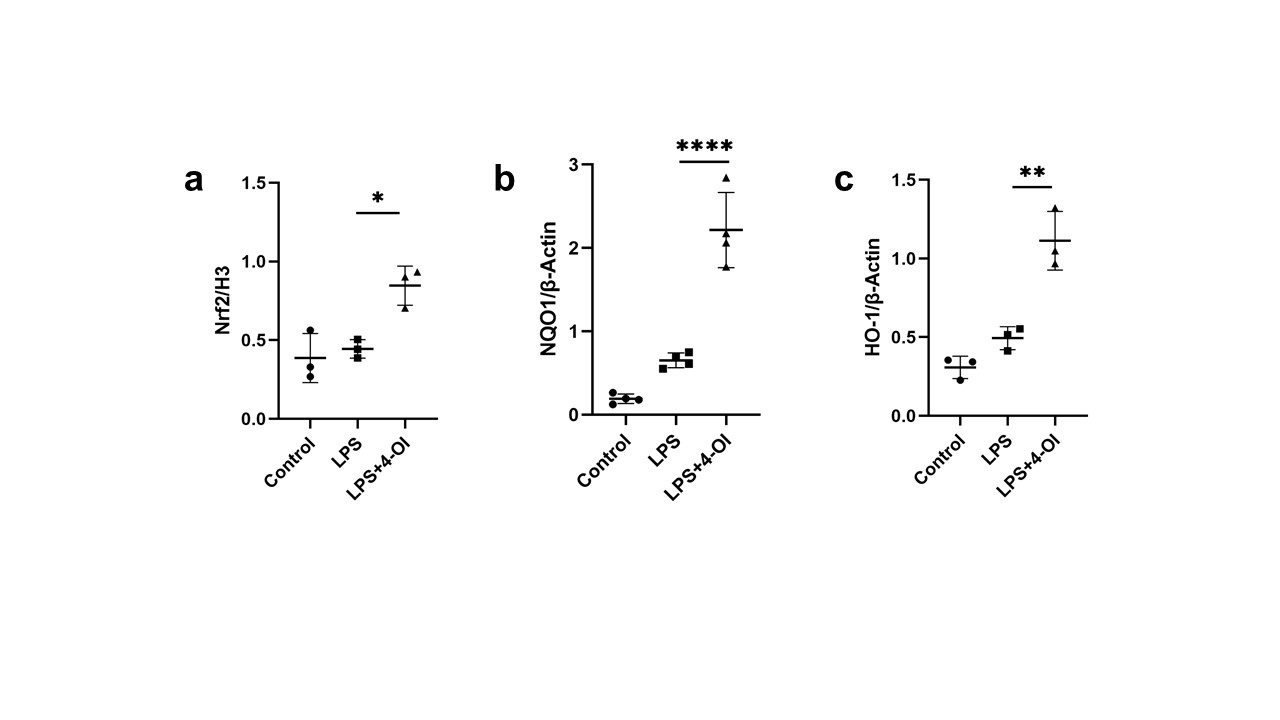


Figure S9: The quantitative expressions of Nrf2 (a) in nuclei fraction lysates, NQO1 (b) and HO-1(c) in cytosol fraction lysates. Data are presented as the mean ± SD. ^*^*p*<0.05; ^**^*p*<0.01; ^****^*p*<0.0001.


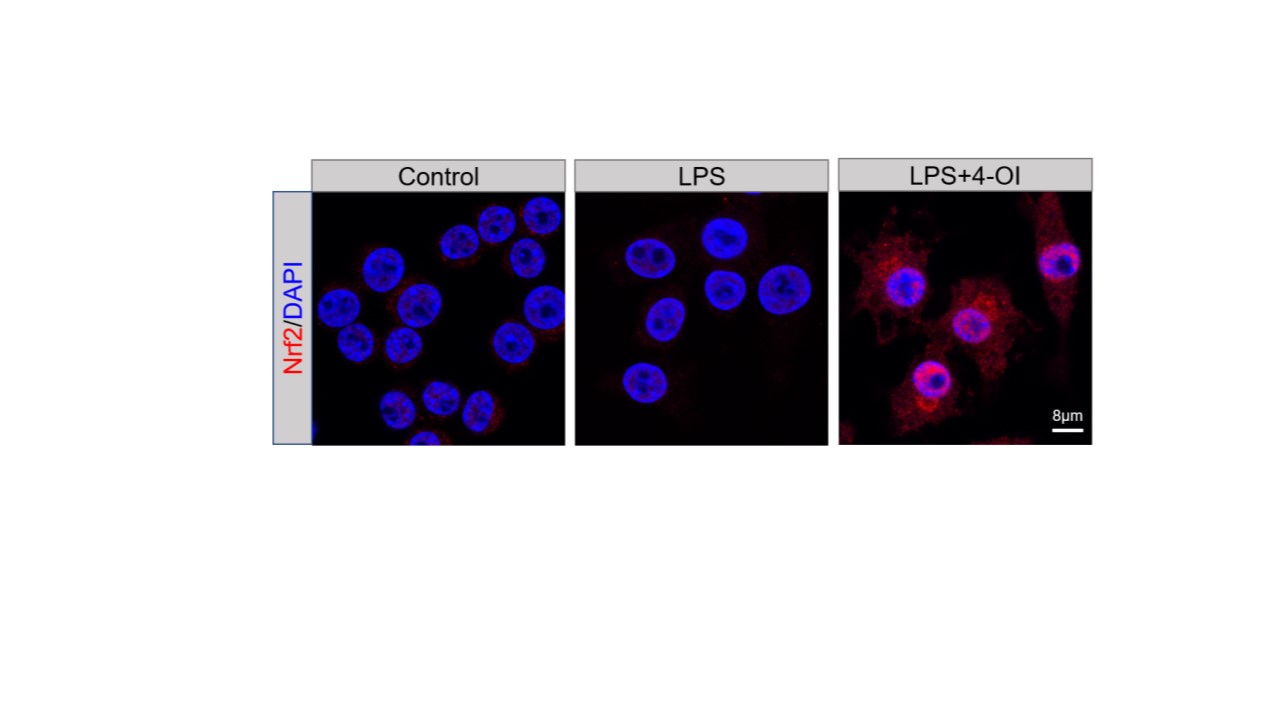


Figure S10: Images of nuclear translocation of Nrf2 detected by IF staining. (Nucleus: blue, Nrf2: red. Scale bar = 8 μm).


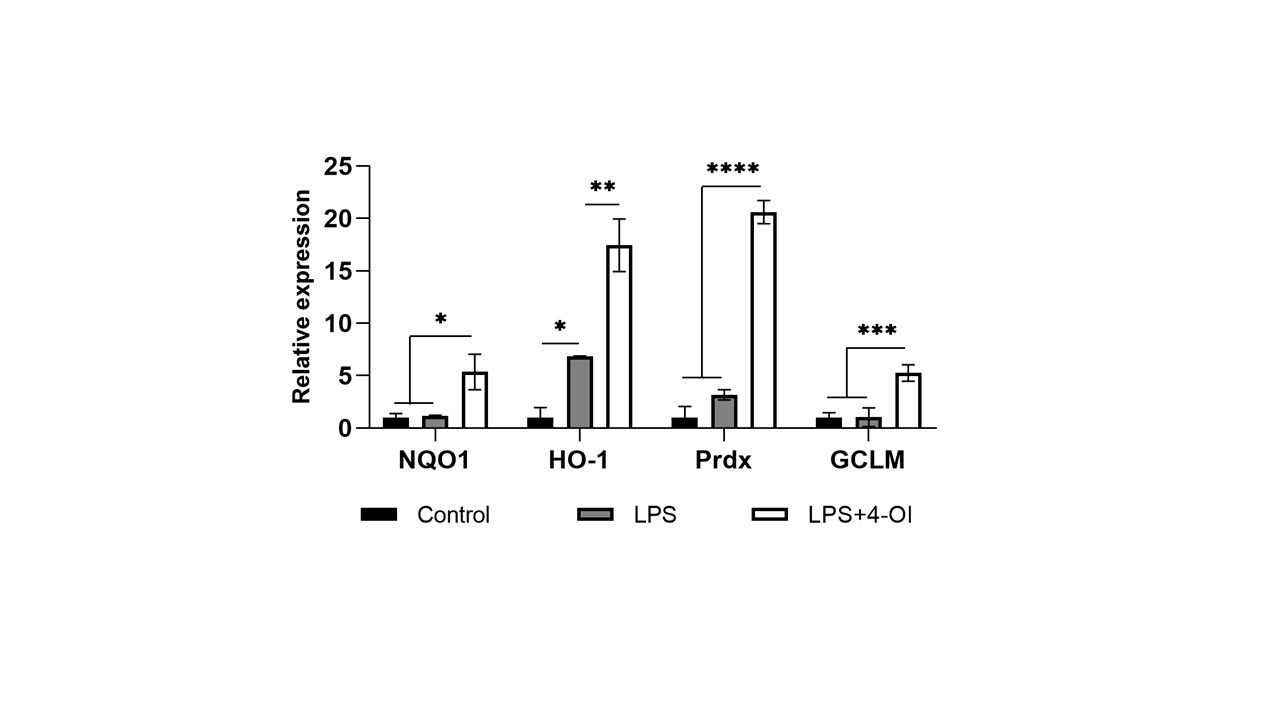


Figure S11: qRT-PCR analysis of NQO1, HO-1, Prdx and GCLM in BMDMs.


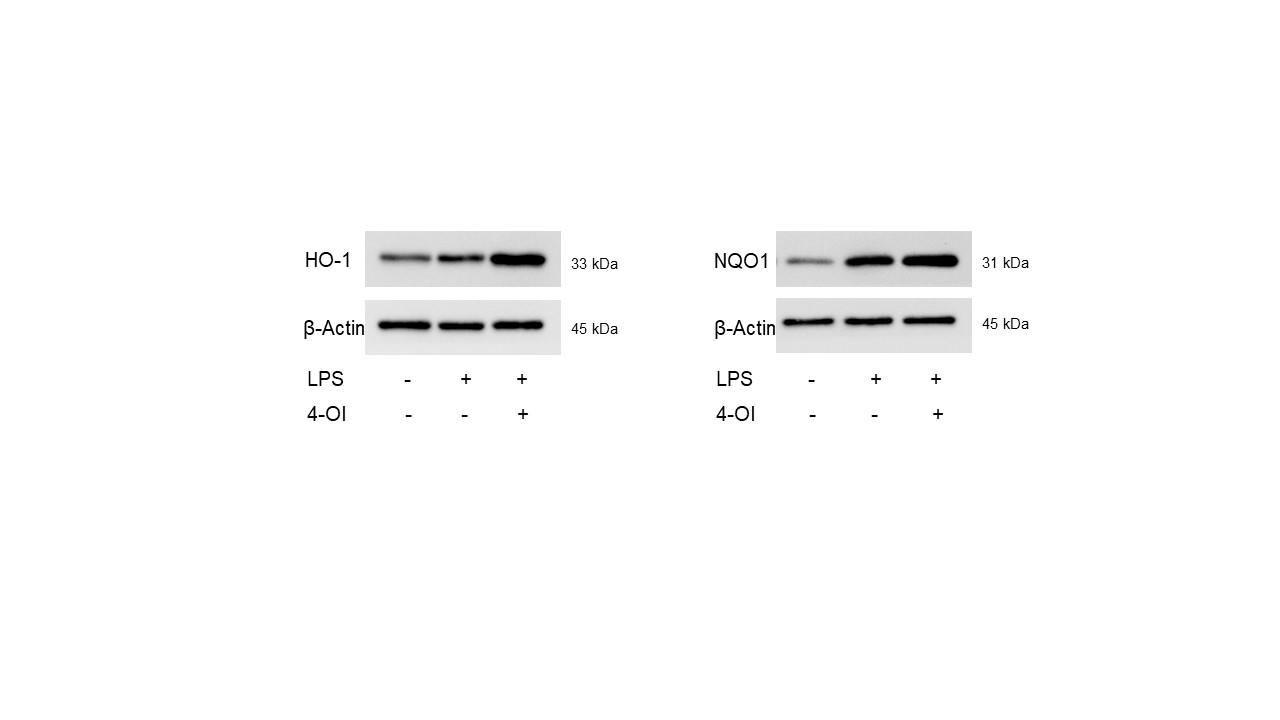


Figure S12: The expression of Nrf2 downstream ARE-dependent proteins in BMDMs measured by western blotting.


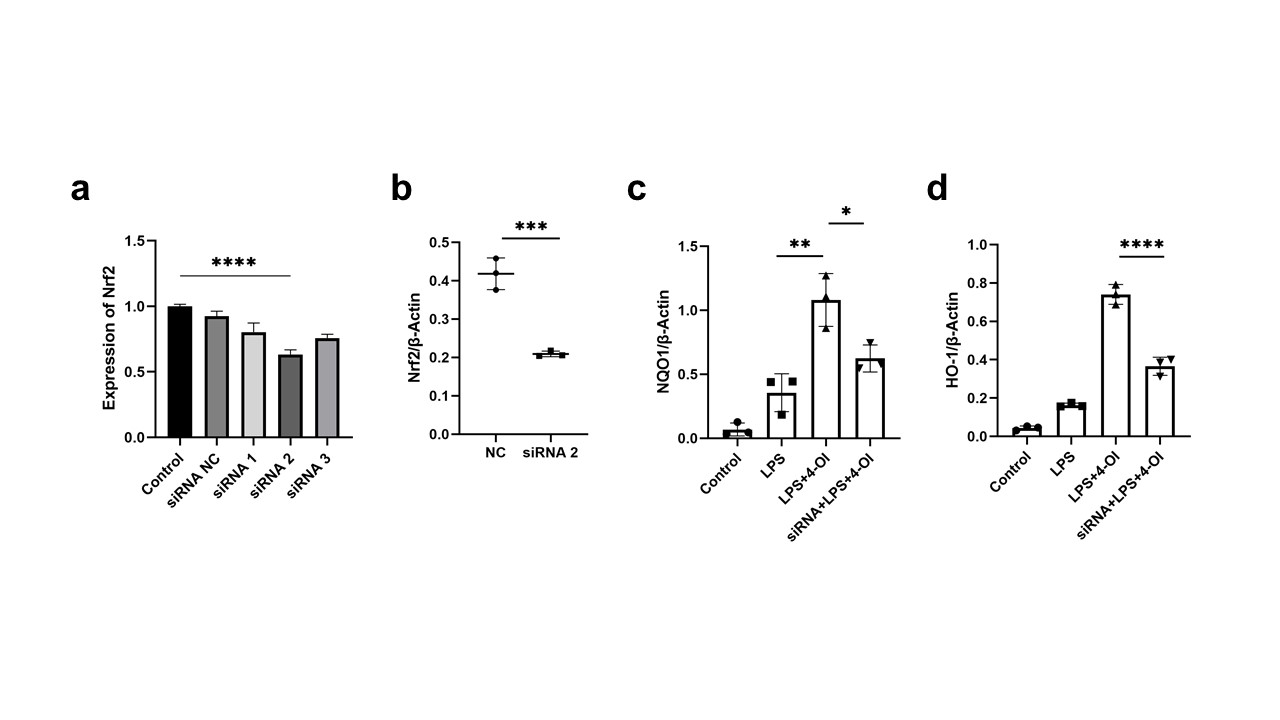


Figure S13: (a) Transfection effects of siRNA or negative control siRNA (NC) analyzed by qRT-PCR. The expressions of Nrf2 (b), NQO1 (c) and HO-1 (d) in cytosol fraction lysates. Data are presented as the mean ± SD. ^*^*p*<0.05; ^**^*p*<0.01; ^***^*p*<0.001; ^****^*p*<0.0001.


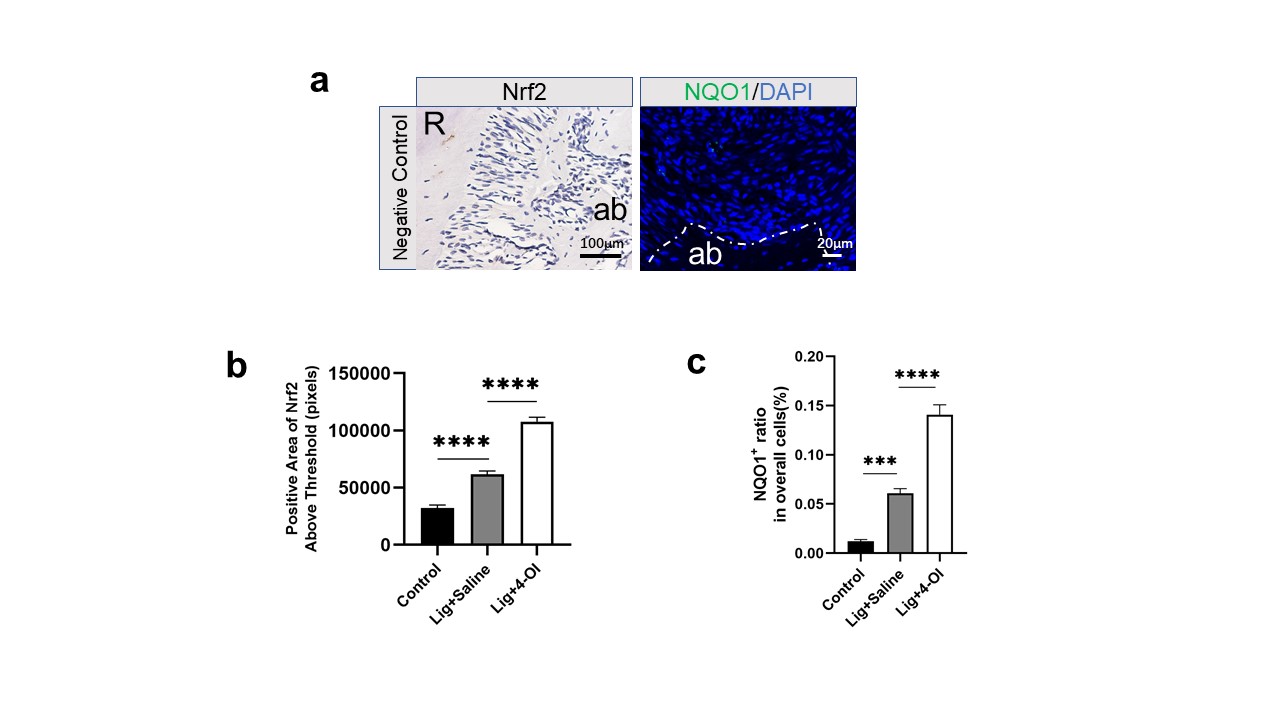


Figure S14: (a) Negative controls of Nrf2 and NQO1 tested by IHC and IF. (b) The semi-quantification of IHC staining of Nrf2. (c) The semi-quantification of IF staining of NQO1. ab: alveolar bone; R: root. (Scale bar = 100, 20 μm)


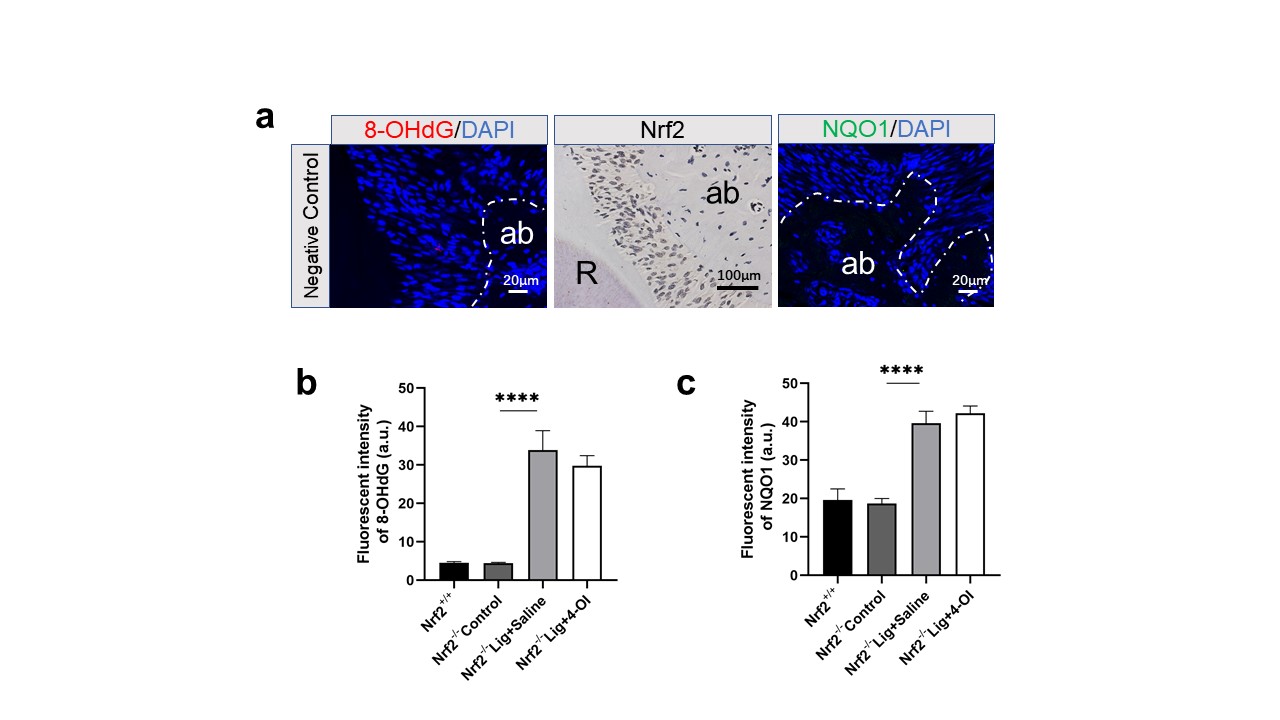


Figure S15: (a) Negative controls of 8-OHdG, Nrf2 and NQO1 tested by IHC and IF. (b, c) Semi-quantitative analysis of fluorescent intensity of 8-OHdG and NQO1. Data are presented as the mean ± SD. ^****^*p*<0.0001. ab: alveolar bone; R: root. (Scale bar = 20, 100 μm)

**Supplementary Table captions:**

Table S1: Prime sequences of mRNA

| Gene | Forward Primer (5'-3’) | Reverse Primer (5'-3’) |
| --- | --- | --- |
| IL-1β | TGAATTGGTCATAGCCCGCA | TCTCCTTCCTGTGCAAACTCT |
| IL-6 | TCCTACCCCAATTTCCAATGCT | AACGCACTAGGTTTGCCGAG |
| CCL2 | AACTGCATCTGCCCTAAGGT | AGGCATCACAGTCCGAGTCA |
| TGF-β | CTTCAGCCTCCACAGAGAAGAACT | TGTGTCCAGGCTCCAAATATAG |
| iNOS | GGCCACCAAGCTGAACTTGA | GTTCCAGCTTCTGGCACTGA |
| CD86 | CTTACGGAAGCACCCACGAT | CGGCAGATATGCAGTCCCAT |
| CD206 | AGACGAAATCCCTGCTACTG | CACCCATTCGAAGGCATTC |
| Arg1 | GGAATCTGCATGGGCAACCTGTGT | AGGGTCTACGTCTCGCAAGCCA |
| HO-1 | CTGTCCAGTTGGTGTGGATAA | TCAGGCAGAGGGTGATAGAA |
| NQO1 | CAGTCAAATCTGGTGGCATC | GCTGCAGACCTGGTGATATT |
| GCLM | GCCATCTCCACAGCAATGTA | GACAAAACACAGTTGGAACAGC |
| Prdx | TGTTCATGAGTTCCACGCCA | CTGCTTCCTGGGCGTATTGA |
| GAPDH | TGAGGTGACCGCATCTTCTTG | TGGTAACCAGGCGTCCGATA |

Table S2: Nrf2 siRNA sequences used in this study

| Name | Sense (5'-3’) | Antisense (5'-3’) |
| --- | --- | --- |
| Strand 1 | AGACAUAGAUCUUGGAGUAdTdT | UACUCCAAGAUCUAUGUCUdTdT |
| Strand 2 | GAAUUACAGUGUCUUAAUAdTdT | UAUUAAGACACUGUAAUUCdTdT |
| Strand 3 | CGUGAGUCCUGGUCAUCAAdTdT | UUGAUGACCAGGACUCACGdTdT |
| Negative control | UUCUCCGAACGUGUCACGUdTdT | ACGUGACACGUUCGGAGAAdTdT |

Table S3: The up-regulation protein layout of cytokine array

| Number | 1 | 2 | 3 | 4 | 5 | 6 | 7 | 8 |
| --- | --- | --- | --- | --- | --- | --- | --- | --- |
| Cytokine | CXCL10 | TIMP-1 | G-CSF | GM-CSF | IL-6 | CCL2 | CCL5 | IL-27 |
